# S100A9 Mediates Neuroblastoma‐Associated Skeletal Muscle Atrophy via O‐GlcNAcylation Repression and Activate Mitophagy Activation

**DOI:** 10.1002/jcsm.70419

**Published:** 2026-11-05

**Authors:** Jianwei Lin, Yuxin Zhang, Qi Wang, Jingchun Lv, Bingqiang Han, Dapeng Jiang

**Affiliations:** ^1^ Department of Oncology (Ward 2), Shanghai Children's Medical Center Shanghai Jiao Tong University School of Medicine Shanghai China; ^2^ Department of Anesthesiology, Shanghai Children's Medical Center Shanghai Jiao Tong University School of Medicine Shanghai China; ^3^ Shanghai Key Laboratory for Nucleic Acid Chemistry and Nanomedicine, Institute of Molecular Medicine, State Key Laboratory of Systems Medicine for Cancer, Shanghai Cancer Institute Renji Hospital, School of Medicine, Shanghai Jiao Tong University Shanghai China; ^4^ Department of Pediatric Orthopedics, Shanghai Children's Medical Center Shanghai Jiao Tong University School of Medicine Shanghai China

**Keywords:** mitophagy, muscle, neuroblastoma, O‐GlcNAcylation, S100A9

## Abstract

**Background:**

In patients with neuroblastoma (NB), skeletal muscle atrophy is a key factor linked to poor survival and decreased long‐term quality of life. Nevertheless, there are still insufficient therapeutic methods that work, and the underlying mechanisms remain unclear. As persistent inflammation is a hallmark of tumour‐related muscle atrophy, this study aims to identify the key inflammatory mediators causing NB‐related muscle atrophy and systematically elucidate the downstream molecular mechanism.

**Methods:**

The adverse biological features of NB were used to identify potential inflammatory mediators causing muscle atrophy. Serum samples from NB patients and NB mouse models were then used to confirm the clinical relevance of these candidates to skeletal muscle atrophy. Recombinant inflammatory proteins and certain inhibitors were given in vivo to define the functional impact of mediators. Additionally, RNA sequencing (RNA‐seq), quantitative real‐time PCR (qPCR) and Western blotting were used to methodically examine the underlying molecular pathways of NB‐induced muscle atrophy.

**Results:**

Both NB patients with muscle atrophy and NB mouse models had considerably higher serum levels of the pro‐inflammatory mediator S100A9 protein (*p* < 0.001). Recombinant mouse S100A9 protein functionally reduced skeletal muscle weight and upregulated the atrophy markers TRIM63/FBXO32 in mice (*p* < 0.05), whereas its inhibitor, ABR‐238901, successfully reduced muscle atrophy and downregulated these atrophy markers in the NB model (*p* < 0.001). Mechanistically, S100A9 inhibited skeletal muscle protein O‐GlcNAcylation via its receptor RAGE (*p* < 0.05). Muscle atrophy was ultimately caused by the abnormal activation of BNIP3/BNIP3L‐mediated mitophagy that resulted from this suppression. Lastly, by inhibiting this mitophagy route, in vivo administration of the O‐GlcNAcylation agonist Thiamet G considerably reduced skeletal muscle atrophy in NB mice (*p* < 0.05).

**Conclusions:**

Our study identifies S100A9 as a critical mediator of NB‐associated skeletal muscle atrophy through a novel RAGE/O‐GlcNAcylation/mitophagy axis, establishing S100A9 as a potential therapeutic target and providing a rationale for early interventional strategies to preserve muscle mass and improve patient outcomes.

## 1 Introduction

While advances in high‐risk neuroblastoma (NB) therapy have raised the 5‐year survival rate to over 50% and expanded the long‐term survivor cohort, tumour‐ and treatment‐related adverse effects substantially compromise patients' long‐term quality of life [1, 2]. For instance, skeletal muscle atrophy, observed in more than 4% of NB survivors, drives chronic fatigue, functional impairment and poor quality of life [2, 3]. Beyond impairing quality of life, skeletal muscle atrophy in NB patients can adversely influence prognosis and treatment responses [3, 4]. Despite growing clinical awareness, the mechanisms driving NB‐related skeletal muscle atrophy remain elusive. This study therefore investigates its pathogenesis.

Systemic inflammation is a key driver of cancer‐related skeletal muscle atrophy [5]. Inflammatory mediators such as IL‐6 and TNFα have been proven to induce atrophy of skeletal muscle [6, 7]. However, IL‐6 and TNF‐α, among others, did not change significantly during NB progression [8]. This contradiction points to other inflammatory mediators that fluctuate during NB progression, triggering muscle atrophy. S100A9, a proinflammatory calcium‐binding protein, is highly expressed in the bone marrow metastatic NB microenvironment [9]. Elevated in the serum of high‐risk NB patients and predicting poor prognosis, S100A9 can drive proliferation, migration and invasion of NB cells [10, 11]. These aggressive phenotypes, together with its poor prognostic implication, led us to hypothesize S100A9 as a key instigator of NB‐associated skeletal muscle atrophy.

Skeletal muscle atrophy is fundamentally driven by an imbalance in protein turnover, favouring degradation over synthesis [12]. Post‐translational modifications (PTMs) play pivotal roles in regulating protein stability and function [13]. O‐linked N‐acetylglucosamine (O‐GlcNAc) modification (O‐GlcNAcylation) represents a dynamic and reversible PTM involving the attachment of a single N‐acetylglucosamine moiety to serine or threonine residues on target proteins [14]. Preserving O‐GlcNAcylation homeostasis protects skeletal muscle from atrophy [15]. Nevertheless, its specific changes in NB‐induced muscle atrophy remain poorly understood.

In this study, we investigated the S100A9/RAGE axis and its downstream effects on O‐GlcNAcylation and mitophagy. Our findings reveal a novel mechanism whereby S100A9 drives muscle wasting via RAGE‐mediated O‐GlcNAcylation inhibition and subsequent BNIP3/BNIP3L‐dependent mitophagy. This establishes S100A9 as a therapeutic target and provides a mechanistic basis for preserving muscle mass in NB.

## 2 Materials and Methods

### 2.1 Cell Culture

C2C12 (FH0345, Shanghai Fuheng Biotechnology, China) and Neuro‐2a (FH0424, Shanghai Fuheng Biotechnology, China) cells were maintained in DMEM (PM150210, Wuhan Pricella Biotechnology, China). Both cell lines were authenticated via STR profiling and tested negative for contamination. The medium was supplemented with 10% foetal bovine serum (FSP500, Excell Biotechnology, China) and 1% penicillin–streptomycin (PB180120, Wuhan Pricella Biotechnology, China), and cells were incubated at 37°C with 5% CO_2_. For C2C12 differentiation into myotubes, growth medium was replaced with DMEM containing 2% horse serum (164215, Wuhan Pricella Biotechnology, China) for 6 days.

### 2.2 Animals and Subcutaneous Tumour Model

Animal procedures followed the NIH Guide for the Care and Use of Laboratory Animals. The research protocol was approved by the Welfare and Ethics Committee of Laboratory Animals of Shanghai SINOGENE Life Science and Technology (Ethics Approval No. XNG201‐2402‐001). Six‐week‐old male Balb/c mice were procured from SPF Biotechnology Co. Ltd. and maintained under specific pathogen‐free (SPF) conditions. Mice were housed in micro‐isolation cages under a 12‐h light/dark cycle with ad libitum access to food and water, whereas ambient temperature and humidity were controlled according to international standards.

Mice received subcutaneous injections of 5 × 10^5^ Neuro‐2a cells into the right axilla. After inoculation, we measured individual food intake by subtracting daily residual diet from the supplied amount and recorded body weight and tumour volume every 2 days. We assessed grip strength using a rat and mouse grip tester (Beijing Zhongshi Dichuang Technology Development Co. Ltd). Mice were euthanized when body weight loss reached 5% of the maximum recorded weight according to humane endpoint criteria. At sacrifice, we excised, weighed and photographed bilateral gastrocnemius and quadriceps muscles for morphological analysis.

### 2.3 Paediatric Muscle Atrophy Assessment and Serum Collection

The subjects of this study included NB patients diagnosed and treated at Shanghai Children's Medical Center from 2016 to 2024. The inclusion criteria were as follows: (1) NB confirmed by pathology; (2) complete clinical data, including imaging, tumour metastasis, risk stratification and MYCN gene amplification status; and (3) age between 1 and 16 years old. Exclusion criteria included: (1) lack of necessary imaging data; (2) excluded secondary infections, including those caused by NB growth compressing the lung and other organs; and (3) the presence of other malignant tumours. The clinical data of 143 patients with pathologically confirmed NB were collected. CT image data were mainly used to measure the total psoas major muscle area (tPMA) at the level of the third (L3) and fourth (L4) lumbar vertebrae. The Paediatric Total Psoas Muscle Area Z‐scores Calculator (https://ahrc‐apps.shinyapps.io/sarcopenia/) was employed to compute individual Z‐score, which served as an objective indicator for assessing skeletal muscle atrophy. Furthermore, pretreatment blood samples were collected from 34 patients, and human skeletal muscle tissues from partial abdominal incisions were removed from 12 patients with NB during tumour resection surgery.

This study was conducted in strict accordance with the ethical principles outlined in the Declaration of Helsinki. The collection and analysis of all human clinical data and samples involved were reviewed and approved by the Ethics Committee/Institutional Review Board of the Shanghai Children's Medical Center (SCMC), Shanghai Jiao Tong University School of Medicine (Approval Nos.: SCMCIRB‐K2023128‐1). Informed consent was obtained from all participants' parents or legal guardians.

Detailed descriptions of additional experimental methods are provided in the Supplementary Information.

### 2.4 Statistical Analysis

Data are expressed as mean ± SD. We used two‐sided unpaired Student *t*‐tests for two group comparisons unless otherwise stated. One way ANOVA with Dunnett post hoc test compared multiple groups (*n* ≥ 3). Spearman correlation assessed relationships between variables, with correlation strength increasing as the absolute R value approached 1. We calculated the optimal tPMA Z‐score cutoff using the R package maxstat, setting group proportions between > 25% and < 75%. The cutoff was −2.09, stratifying patients into high and low groups. The survfit function from the R package survival estimated survival, and log rank tests evaluated prognostic differences. The significance level is defined as follows: *p < 0.05, **p < 0.01, ***p < 0.001 and ****p < 0.0001. We performed all analyses in GraphPad Prism (v10.0), supplemented by online tools where indicated.

## 3 Results

### 3.1 Elevated Serum S100A9 Correlates With Skeletal Muscle Atrophy in NB Patients

Kaplan–Meier survival curves revealed significantly shorter overall survival (OS) in NB patients with tPMA Z‐score below −2.09 versus those above this cutoff (Figure 1A). Low tPMA Z‐scores also correlated with risk stratification and tumour bone marrow metastasis. Specifically, patients at high risk or with bone marrow metastasis exhibited lower tPMA Z‐scores than those without these features (Figure 1B).

**FIGURE 1:**
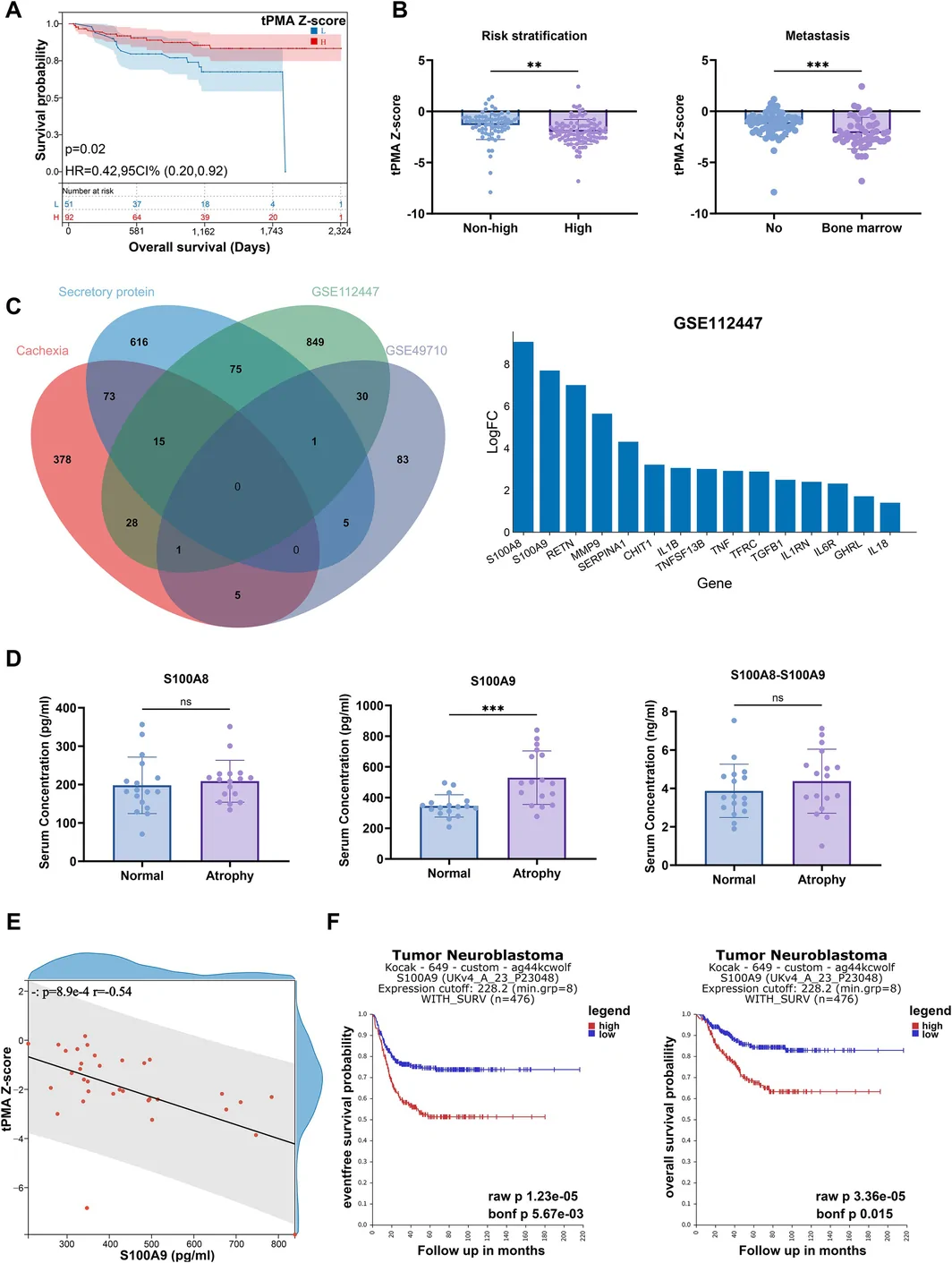
NB‐related skeletal muscle atrophy is associated with S100A9. (A) With a cutoff value of −2.09 for the tPMA Z‐score, the survival curves of patients with NB in different groups. (B) Comparative analysis of tPMA Z‐score between high‐risk and non‐high‐risk NB patients, as well as between NB patients with and without bone marrow metastasis. (C) Venn diagram and subsequent analysis of intersecting genes. The diagram identifies genes common to genes highly expressed in NB with bone marrow metastasis (dataset GSE112447), genes highly expressed in high‐risk NB (dataset GSE49710), cachexia‐related genes from GeneCards and genes related to blood‐secretory proteins from The Human Protein Atlas. The bar graph displays the intersecting genes from GSE112447, ranked in descending order of Log_2_ fold change (LogFC). (D) With a cutoff value of −2.09 for the tPMA Z‐score, serum levels of S100A8, S100A9 and the S100A8/A9 heterodimer in NB patients across different groups were measured by ELISA. (E) Spearman correlation analysis of serum S100A9 protein content and tPMA Z‐score in NB patients. (F) Kaplan–Meier survival curves derived from the R2 database, demonstrating the association between S100A9 expression and overall survival as well as event‐free survival in NB patients. Values are mean ± SD. Significance determined using unpaired *t*‐test. *p < 0.05, **p < 0.01, ***p < 0.001 and ****p < 0.0001.

Based on the association between NB‐associated skeletal muscle atrophy and these adverse tumour clinical features, genes related to cachexia and secreted proteins were screened from the NB tumour highly expressed genes in bone marrow metastasis (GSE112447) and high‐risk NB tumour highly expressed genes (GSE49710) (Figure 1C). S100A9 and S100A8, cachexia‐related secretory protein genes, were the most differentially expressed in NB tumours with bone marrow metastasis, whereas high‐risk NB showed no detectable expression of any such genes (Figure 1C). Serum S100A9 was significantly elevated in NB patients with skeletal muscle atrophy and inversely correlated with the tPMA Z‐scores (Figure 1D,E), whereas S100A8 and the S100A8‐S100A9 dimer showed no significant change (Figure 1D). Beyond its link to skeletal muscle atrophy, high S100A9 expression also predicted poor event‐free survival and OS in NB patients (Figure 1F). These findings suggest that S100A9 not only forecasts poor prognosis but also acts as a mediator of NB‐related skeletal muscle atrophy.

### 3.2 S100A9 Drives Muscle Atrophy in NB Mice

Elevated S100A9 in NB patients with muscle atrophy prompted us to construct an NB mouse model for validation. During tumour progression, NB mice showed significantly lower body weight and grip strength than controls (Figure 2A). Food intake did not differ between groups (Figure S1A,B), ruling out its contribution to skeletal muscle atrophy. Blood S100A9 levels were significantly higher in NB mice and negatively correlated with body weight (Figure 2B). Further analysis revealed reduced gastrocnemius and quadriceps mass and smaller muscle fibre cross‐sectional area (Figures 2C,D and S2A,B). At the molecular level, atrophy‐related proteins TRIM63 and FBXO32 were upregulated in these muscles (Figures 2E and S2C). Thus, the NB model develops skeletal muscle atrophy concurrent with increased circulating and local S100A9.

**FIGURE 2:**
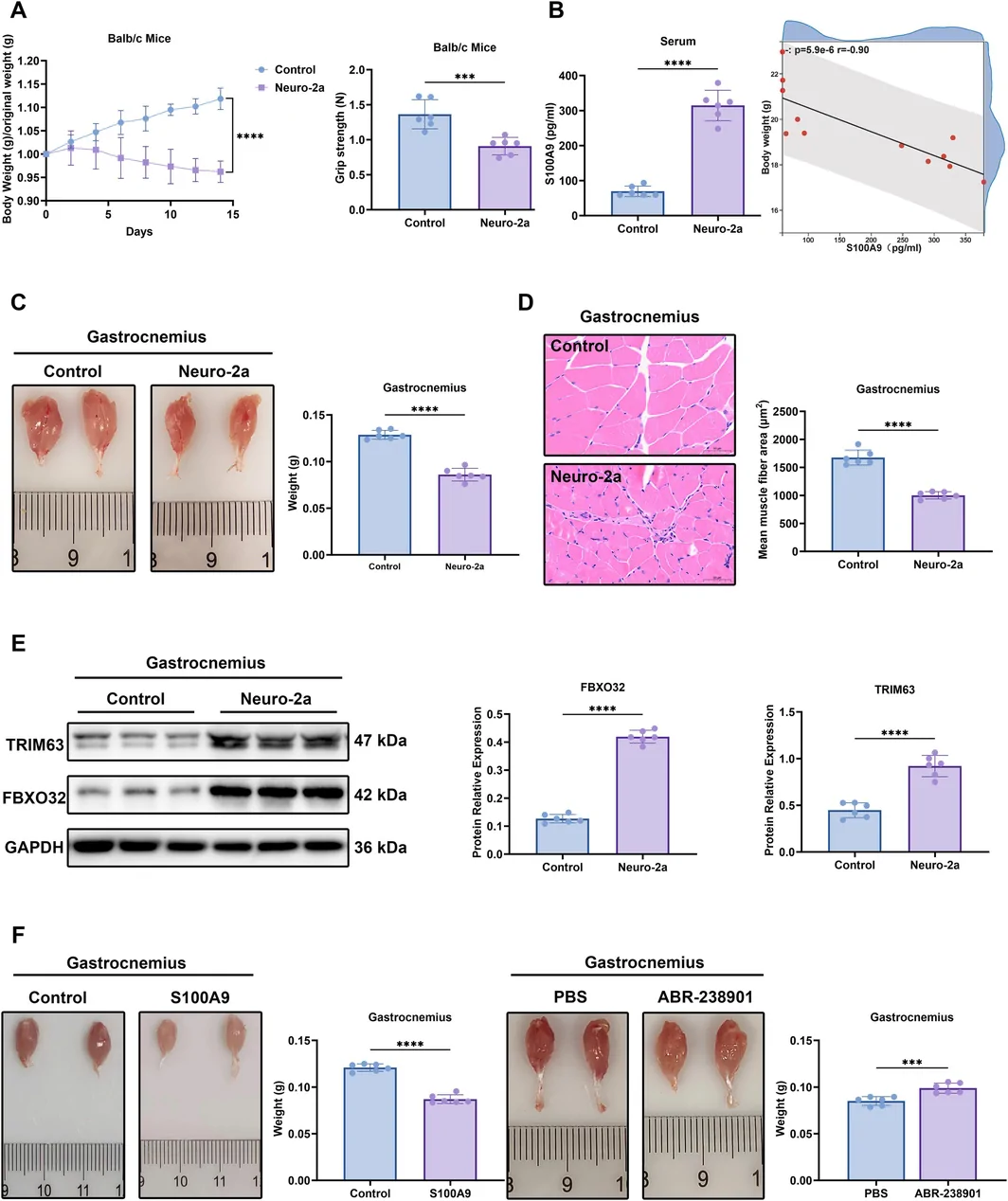
S100A9‐mediated NB‐associated skeletal muscle atrophy. (A) Comparison of weight change curves and grip strength between the NB mouse model and the control group mice (*n* = 6 per group). (B) Comparative analysis of serum S100A9 levels between the NB mouse model and its control mice (*n* = 6 per group), and spearman correlation analysis between serum S100A9 levels and mouse body weight. (C) Gastrocnemius muscle images and weight quantification from NB mouse model and its control mice (*n* = 6 per group). (D) Representative HE‐stained cross‐sectional images of gastrocnemius muscles and quantification of mean muscle fibre area of muscle fibres from NB mouse model and control mice (*n* = 6 per group). Scale bar: 50 μm. (E) Protein expression levels and grayscale quantification of TRIM63 and FBXO32 in gastrocnemius muscles of NB mouse model and control mice (*n* = 6 per group). (F) Gastrocnemius muscle images and weight quantification from mice treated with recombinant mouse S100A9 protein (20 μg/mouse; *n* = 6) and PBS control (*n* = 6), as well as NB mice treated with the S100A9 inhibitor ABR‐238901 (30 mg/kg; *n* = 6) and PBS control (*n* = 6). Values are mean ± SD. Significance determined using unpaired *t*‐test. *p < 0.05, **p < 0.01, ***p < 0.001 and ****p < 0.0001. HE, haematoxylin and eosin.

To test the pathogenic role of S100A9, we injected wild‐type mice with S100A9 recombinant protein and NB mice with the antagonist ABR‐238901. Exogenous S100A9 induced weight loss and grip strength decline in wild‐type mice (Figure S3A), whereas ABR‐238901 alleviated these deficits in NB mice (Figure S3B). S100A9 injection reduced gastrocnemius and quadriceps weight and fibre area and increased TRIM63 and FBXO32 expression (Figures 2F and S4A–D). Conversely, ABR‐238901 increased muscle weight and fibre area and decreased TRIM63 and FBXO32 levels (Figures 2F and S4A–D). These findings establish S100A9 as a key inflammatory mediator of NB‐associated skeletal muscle atrophy and demonstrate that targeted inhibition effectively relieves tumour‐induced muscle wasting.

### 3.3 Reduced O‐GlcNAcylation in NB‐Induced Muscle Atrophy

GSEA of transcriptomes from atrophied gastrocnemius muscle in NB mice revealed cellular biological process changes driven by elevated S100A9. O‐GlcNAcylation was significantly downregulated in atrophied gastrocnemius muscle (Figure 3A). The key enzymes OGT and OGA were downregulated and upregulated, respectively, in the atrophied gastrocnemius muscle (Figure 3B). Protein validation confirmed reduced O‐GlcNAcylation in atrophied skeletal muscle under NB conditions. Figures 3C–E and S5A,B show O‐GlcNAcylation and OGT changes across atrophied gastrocnemius and quadriceps of NB mice, S100A9‐treated mice and atrophied rectus abdominis of NB patients. O‐GlcNAcylation and OGT were significantly downregulated in all atrophied muscles, whereas OGA showed no consistent difference between normal and atrophied tissues.

**FIGURE 3:**
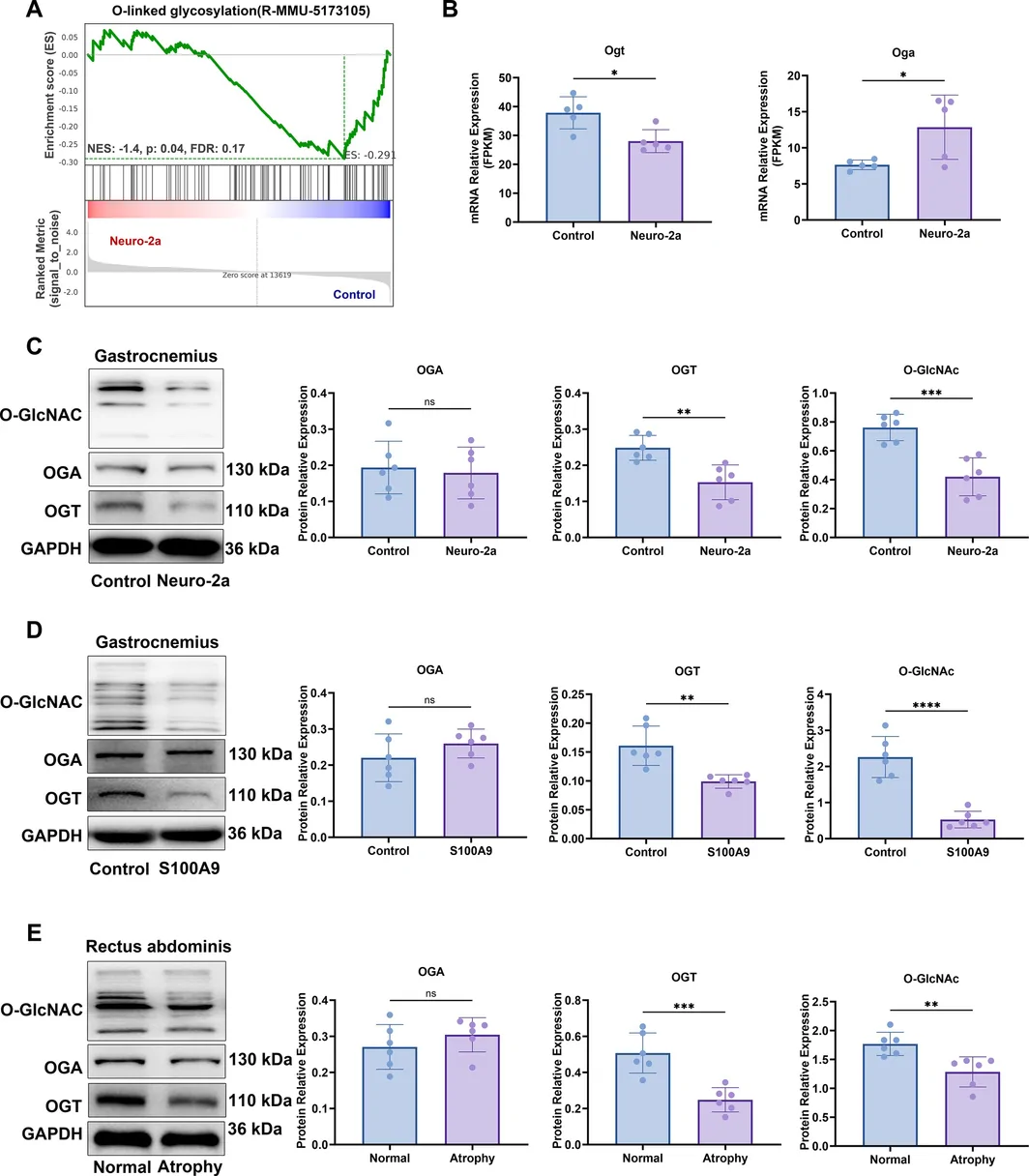
Downregulation of O‐GlcNAcylation in NB‐associated atrophied skeletal muscle. (A) Analysis of the changes of O‐GlcNAcylation modification pathway in the atrophied gastrocnemius muscle of NB mice by Gene Set Enrichment Analysis (GSEA). (B) The expression status of Ogt and Oga genes in the atrophied gastrocnemius muscle in the transcriptome data. (C) Protein expression levels and grayscale quantification of OGT, OGA and overall O‐GlcNAcylation modification level in gastrocnemius muscles from the NB mouse models and control mice (*n* = 6 per group). (D) Protein expression levels and grayscale quantification of OGT, OGA and overall O‐GlcNAcylation modification level in gastrocnemius muscles from the mouse treated with recombinant mouse S100A9 protein (20 μg/mouse; *n* = 6) or PBS control (*n* = 6). (E) Protein expression levels and grayscale quantification of OGT, OGA and overall O‐GlcNAcylation modification level in rectus abdominis muscles from NB patients with or without muscle atrophy (*n* = 6 per group). Values are mean ± SD. Significance determined using unpaired *t*‐test.*p < 0.05, **p < 0.01, ***p < 0.001 and ****p < 0.0001.

### 3.4 O‐GlcNAcylation Downregulation Drives S100A9‐Induced Skeletal Muscle Atrophy

To examine O‐GlcNAcylation downregulation in skeletal muscle atrophy, we manipulated O‐GlcNAcylation levels in C2C12 myotubes using the OGT inhibitor OSMI‐1 and OGA inhibitor Thiamet G. Figure S6A–D show that 20 μM OSMI‐1 and 1 μM Thiamet G effectively altered O‐GlcNAcylation and viability in C2C12 myotubes. OSMI‐1 treatment significantly increased atrophy‐associated proteins TRIM63 and FBXO32 (Figure 4A) and reduced myotube diameter (Figure 4B). S100A9, a key mediator of NB‐associated muscle atrophy, promoted O‐GlcNAcylation at 125 ng/mL but suppressed it at 2000 ng/mL (Figure 4C). Conversely, 1 μM Thiamet G blocked S100A9‐induced reductions in myotube diameter and decreased TRIM63 and FBXO32 expression (Figure 4D–F). These findings confirm that S100A9‐driven O‐GlcNAcylation downregulation is a critical process in muscle atrophy.

**FIGURE 4:**
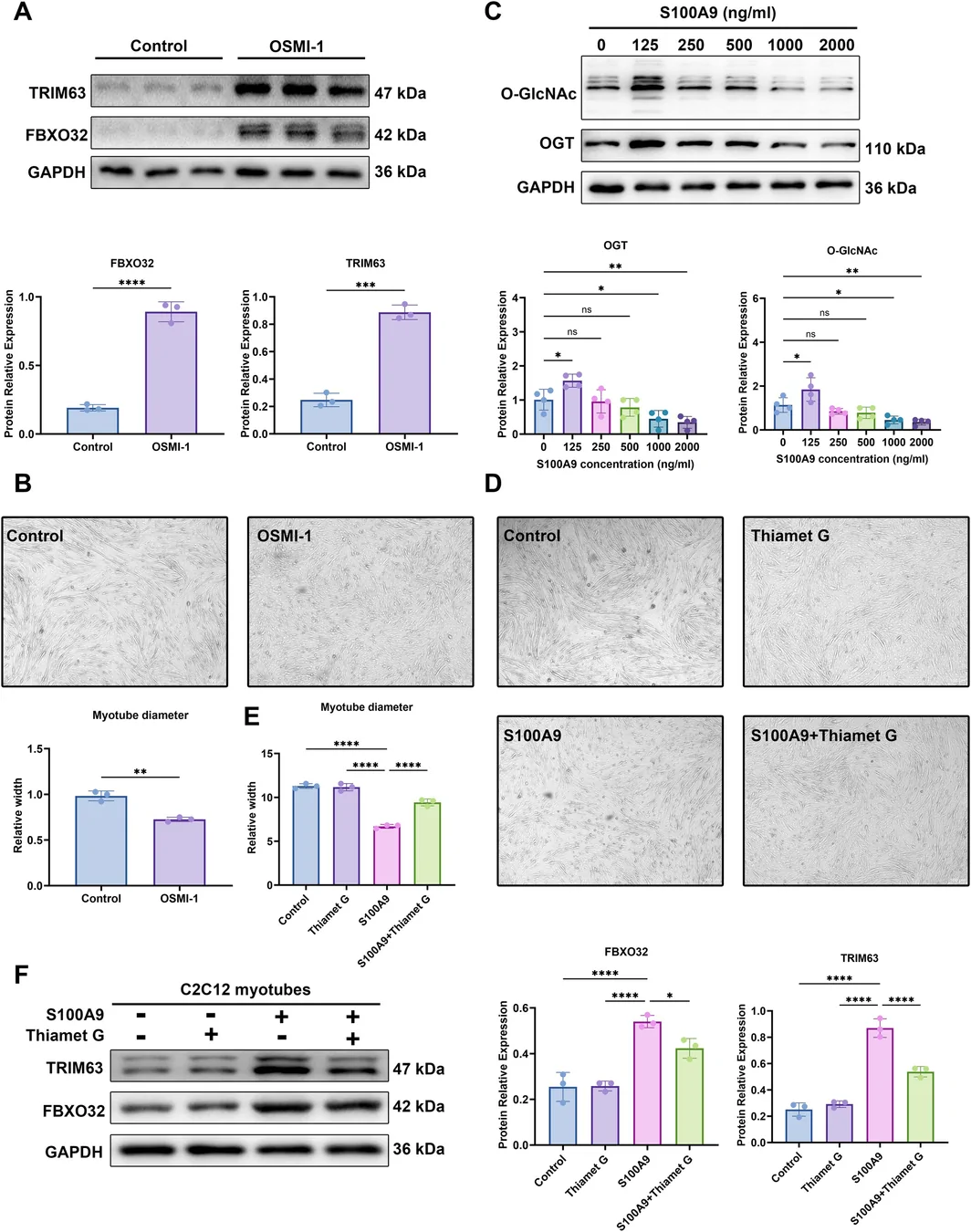
S100A9 induces skeletal muscle atrophy via suppression of O‐GlcNAcylation in C2C12 myotubes. (A) Protein expression levels and grayscale quantification of FBXO32 and TRIM63 in C2C12 myotubes treated with OSMI‐1 (20 μM) or DMSO (*n* = 3 per group). (B) Representative images of C2C12 myotubes following OSMI‐1 (20 μM) or DMSO (vehicle control) treatment, accompanied by quantification of myotube diameter (*n* = 3 per group). Scale bar: 100 μm. (C) Protein expression levels of OGT and overall O‐GlcNAcylation modification level in C2C12 myotubes treated with different concentrations of recombinant mouse S100A9 protein (*n* = 4 per group). (D) Protein expression levels and grayscale quantification of FBXO32 and TRIM63 across four experimental groups: Control (PBS), Thiamet G (1 μM), recombinant mouse S100A9 protein (2000 ng/mL) and the combination of Thiamet G with recombinant mouse S100A9 protein (*n* = 3 per group). (E, F) Representative images of C2C12 myotubes following Control (PBS), Thiamet G (1 μM), recombinant mouse S100A9 protein (2000 ng/mL) and the combination of Thiamet G with recombinant mouse S100A9 protein (*n* = 3 per group). Scale bar: 100 μm. Values are mean ± SD. Significance determined using unpaired *t*‐test and one‐way ANOVA followed by Dunnett's multiple comparisons test for comparisons among three or more groups. *p < 0.05, **p < 0.01, ***p < 0.001 and ****p < 0.0001.

### 3.5 Skeletal Muscle O‐GlcNAcylation Downregulation Triggers Mitophagy

To identify how O‐GlcNAcylation downregulation induces skeletal muscle atrophy, we performed transcriptome sequencing on C2C12 myotubes after OSMI‐1 treatment (Figure 5A). GSEA revealed suppressed O‐GlcNAcylation related processes and activated mitophagy pathways (Figure 5A). Among mitophagy genes, OSMI‐1 upregulated Pink1, Bnip3 and Bnip3l but not Prkn (Figure 5B). Given the stronger response in receptor‐mediated mitophagy genes Bnip3 and Bnip3l, we focused on BNIP3/BNIP3L‐mediated mitophagy.

**FIGURE 5:**
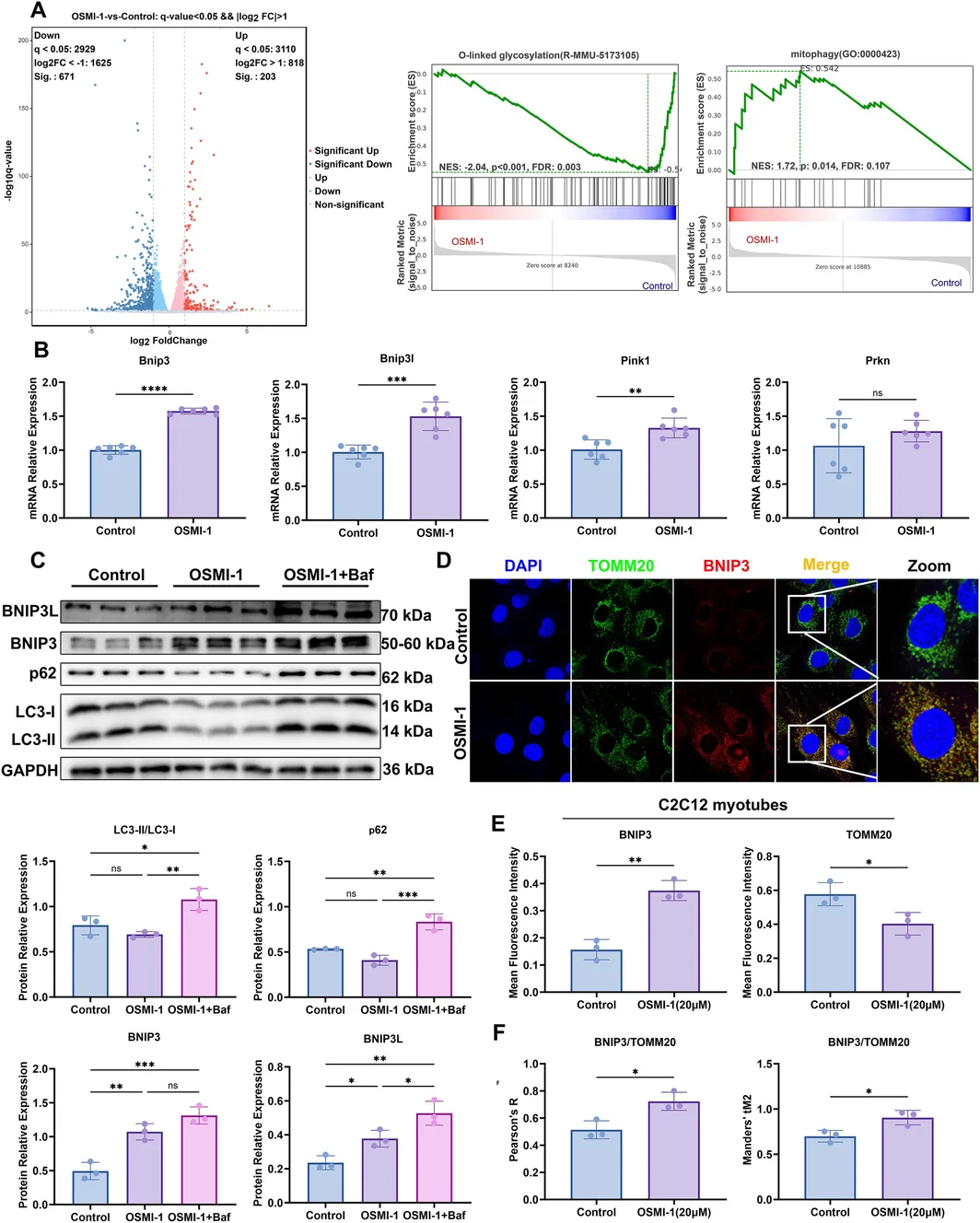
OSMI‐1‐induced activation of receptor‐mediated mitophagy in C2C12 myotubes. (A) Volcano plot of differentially expressed genes and GSEA enrichment analysis for O‐GlcNAcylation and mitophagy pathways in C2C12 myotubes following OSMI‐1 treatment. (B) Gene expression of Bnip3, Bnip3l, Pink1 and Prkn in C2C12 myotubes treated with OSMI‐1 (20 μM) and control (*n* = 6 per group). (C) Protein expression levels and grayscale quantification of BNIP3L, BNIP3, p62, the ratio of LC3‐II to LC‐I in C2C12 myotubes following DMSO (control), OSMI‐1 (20 μM) and the combination of Bafilomycin A1 (Baf, 100 nM) with OSMI‐1 (*n* = 3 per group). (D–F) Representative immunofluorescence images showing the co‐localization of BNIP3 and TOMM20, and quantification of their fluorescence intensity, in C2C12 myotubes treated with OSMI‐1 (20 μM) or DMSO (vehicle control) (*n* = 3 per group). Scale bar: 25 μm. Values are mean ± SD. Statistical significance was determined by an unpaired *t*‐test for comparisons between two groups, and by one‐way ANOVA followed by Dunnett's multiple comparisons test for comparisons among three or more groups. *p < 0.05, **p < 0.01, ***p < 0.001 and ****p < 0.0001.

To verify that O‐GlcNAcylation loss enhances mitophagy, we treated C2C12 myotubes with OSMI‐1 and assessed mitochondrial function. OSMI‐1 reduced mitochondrial membrane potential, indicating impaired function (Figure S7). Co‐treatment with OSMI‐1 and Baf increased BNIP3, BNIP3L, p62 and the LC3‐II/LC3‐I ratio (Figure 5C). Immunofluorescence confirmed reduced mitochondrial TOMM20 and accumulated BNIP3 (Figure 5D,E), with enhanced BNIP3 mitochondrial co‐localization (Pearson and Manders coefficients) after OSMI‐1 treatment (Figure 5F).

S100A9 recombinant protein also induced autophagy and mitophagy (Figure S8). Baf co‐treatment amplified S100A9 induced BNIP3, BNIP3L, p62 and LC3‐II/LC3‐I ratio (Figure S8). To confirm mitophagy as the key mechanism, we knocked down BNIP3 using siRNA. Among three sequences, siBNIP3‐2 was most effective (Figure S9A,B). BNIP3 knockdown reduced S100A9 induced atrophy associated protein expression (Figure S9C) and rescued myotube diameter loss (Figure S9D). In vivo, TEM of atrophied gastrocnemius from NB mice showed mitochondrial vacuolization and autophagosome formation (Figure 6A). Mitophagy proteins BNIP3 and BNIP3L increased, p62 decreased and LC3‐II/LC3‐I ratio increased in gastrocnemius and quadriceps (Figures 6B,C and S10A,B). Similar mitochondrial changes appeared in S100A9 induced atrophied gastrocnemius (Figure 6D). Comparable protein changes occurred in S100A9 treated mouse muscles and atrophied rectus abdominis of NB patients (Figures 6E,F and S10C–F). These findings establish that O‐GlcNAcylation downregulation triggers skeletal muscle atrophy by enhancing receptor‐mediated mitophagy.

**FIGURE 6:**
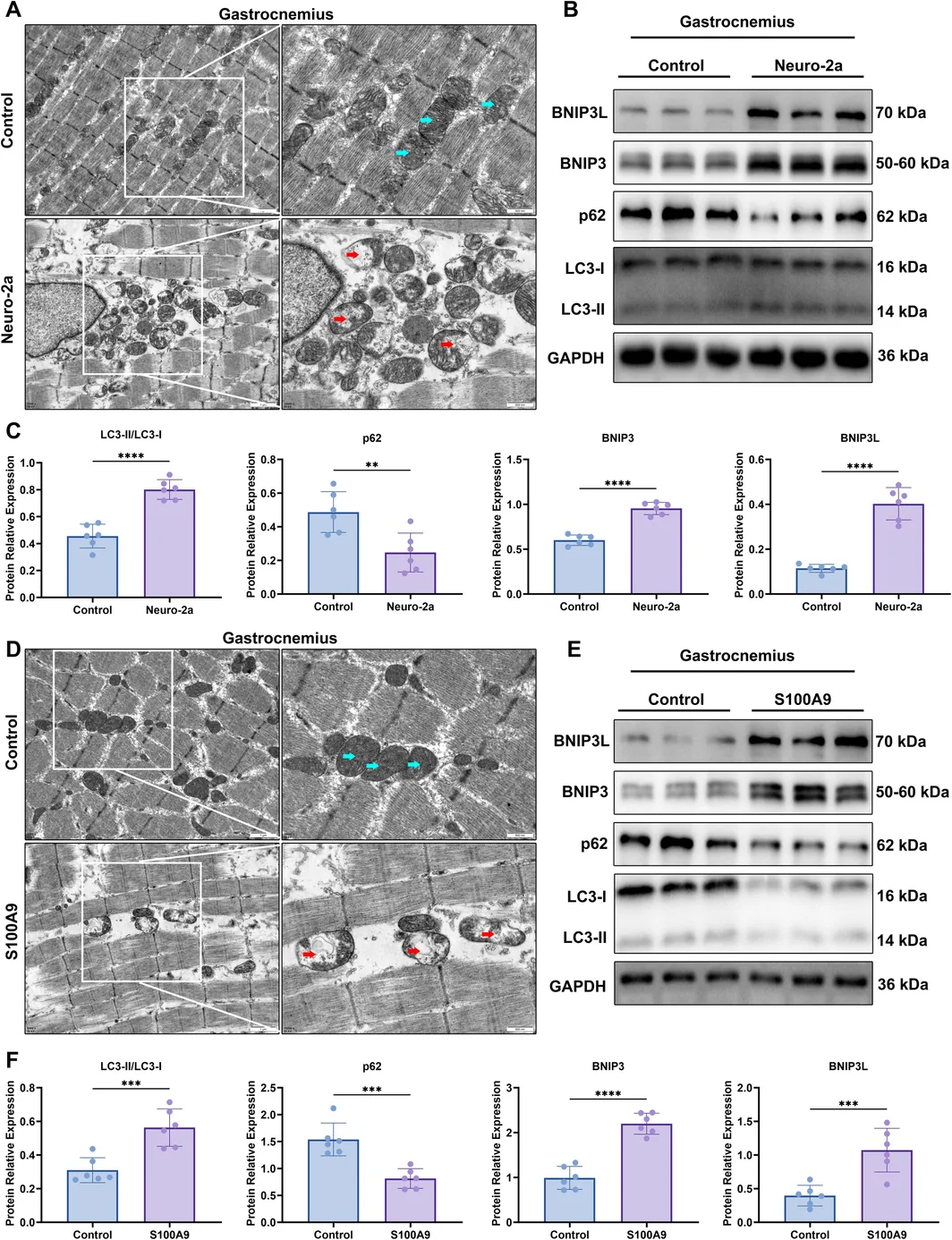
Activation of mitophagy in skeletal muscle atrophy related to NB. (A) Transmission electron microscopy of the gastrocnemius muscle of the NB mouse model and its control mice. Scale bar: 1 μM and 500 nm. (B, C) Protein expression levels and grayscale quantification of BNIP3L, BNIP3, p62, the ratio of LC3‐II to LC‐I in gastrocnemius muscles from the NB mouse models and control group (*n* = 6 per group). (D) Transmission electron microscopy of the gastrocnemius muscle of the mouse treated with recombinant mouse S100A9 protein or PBS. Scale bar: 1 μM and 500 nm. (E, F) Protein expression levels and grayscale quantification of BNIP3L, BNIP3, p62, the ratio of LC3‐II to LC‐I in gastrocnemius muscles from the mouse treated with recombinant mouse S100A9 protein or PBS (*n* = 6 per group). Values are mean ± SD. Significance determined using unpaired *t*‐test. *p < 0.05, **p < 0.01, ***p < 0.001 and ****p < 0.0001.

### 3.6 RAGE Drives NB‐Associated Skeletal Muscle Atrophy

S100A9 receptors include RAGE and TLR4 receptors. To identify the key receptor in S100A9‐induced muscle atrophy, we performed immunofluorescence for RAGE and TLR4 in atrophied rectus abdominis from NB patients and atrophied gastrocnemius from NB mice. In patient samples, TLR4 fluorescence showed no significant increase, whereas RAGE intensity was significantly elevated (Figure 7A,B). In NB mice, both TLR4 and RAGE intensities increased in atrophied gastrocnemius (Figure 7A,B). Protein validation confirmed upregulated RAGE in atrophied rectus abdominis of patients and atrophied gastrocnemius of mice (Figure S11A,B).

**FIGURE 7:**
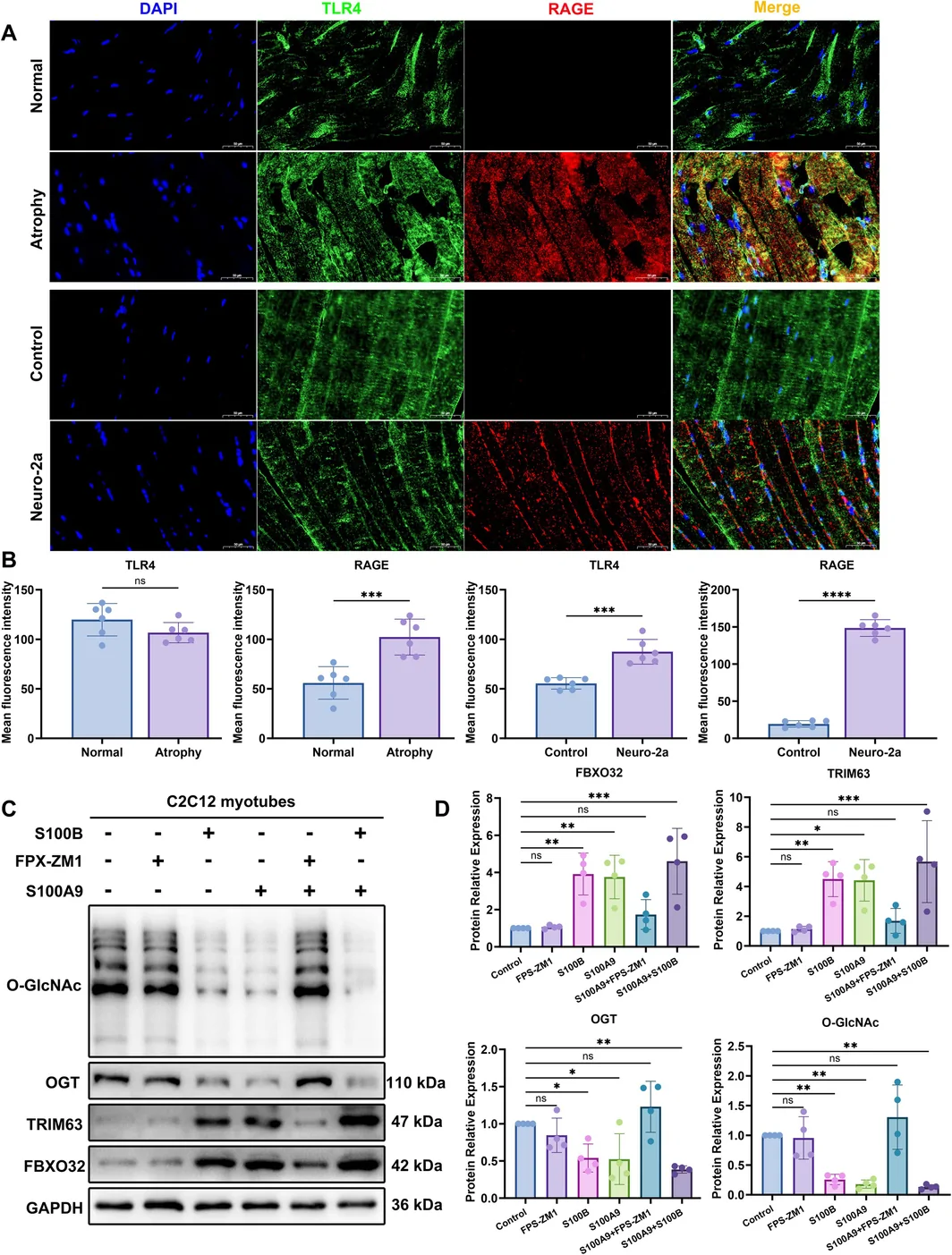
O‐GlcNAcylation in skeletal muscle regulated by S100A9 through RAGE receptor. (A, B) Representative immunofluorescence images and quantification of fluorescence intensity for TLR4 and RAGE receptors in rectus abdominis muscles from NB patients (*n* = 6 per group) and in gastrocnemius muscles from NB mice and their control counterparts (*n* = 6 per group). Scale bar: 50 μm. (C, D) Protein expression levels and grayscale quantification of FBXO32 and TRIM63 across six experimental groups: Control (PBS), FPS‐ZM1 (20 nM), recombinant mouse S100A9 protein (2000 ng/mL), recombinant mouse S100B protein (20 μg/mL), the combination of FPS‐ZM1 with recombinant mouse S100A9 protein and the combination of recombinant mouse S100B protein with recombinant mouse S100A9 protein (*n* = 3 per group). Values are mean ± SD. Statistical significance was determined by an unpaired *t*‐test for comparisons between two groups, and by one‐way ANOVA followed by Dunnett's multiple comparisons test for comparisons among three or more groups. *p < 0.05, **p < 0.01, ***p < 0.001 and ****p < 0.0001.

To determine functional role of RAGE, we treated cells with S100A9 recombinant protein combined with RAGE‐specific agonists or inhibitors. The RAGE agonist S100B promoted atrophy‐related protein expression and suppressed O‐GlcNAcylation and OGT levels (Figure 7C,D). Conversely, the RAGE inhibitor FPS‐ZM1 relieved S100A9‐induced atrophy, restored O‐GlcNAcylation and increased OGT protein content (Figure 7C,D). In vivo, FPS‐ZM1 significantly alleviated weight loss and improved grip performance in NB mice (Figure S12A,B). FPS‐ZM1 treatment also increased gastrocnemius and quadriceps weights and restored muscle fibre cross‐sectional areas (Figure S12C–F). Additionally, FPS‐ZM1 upregulated overall O‐GlcNAcylation and decreased atrophy‐related protein levels (Figure S13A,B). These findings establish RAGE as the key receptor mediating biological effects of S100A9 in NB‐associated skeletal muscle atrophy.

### 3.7 Thiamet G In Vivo Suppresses Mitophagy and Alleviates Skeletal Muscle Atrophy in NB Mice

Figure 8A shows the experimental flow of Thiamet G treatment for skeletal muscle atrophy in NB mice. Thiamet G alleviated weight loss and significantly improved grip strength (Figure 8B). Tumour volume did not differ between groups (Figure S14A,C). Thiamet G also increased the weight and muscle fibre cross‐sectional area of the gastrocnemius and quadriceps (Figure 8C,D). Furthermore, it reduced BNIP3 and BNIP3L expression in these muscles (Figure 8E,F). These findings establish that skeletal muscle O‐GlcNAcylation downregulation is the core process in NB‐induced atrophy, and in vivo modulation of this pathway effectively relieves the phenotype.

**FIGURE 8:**
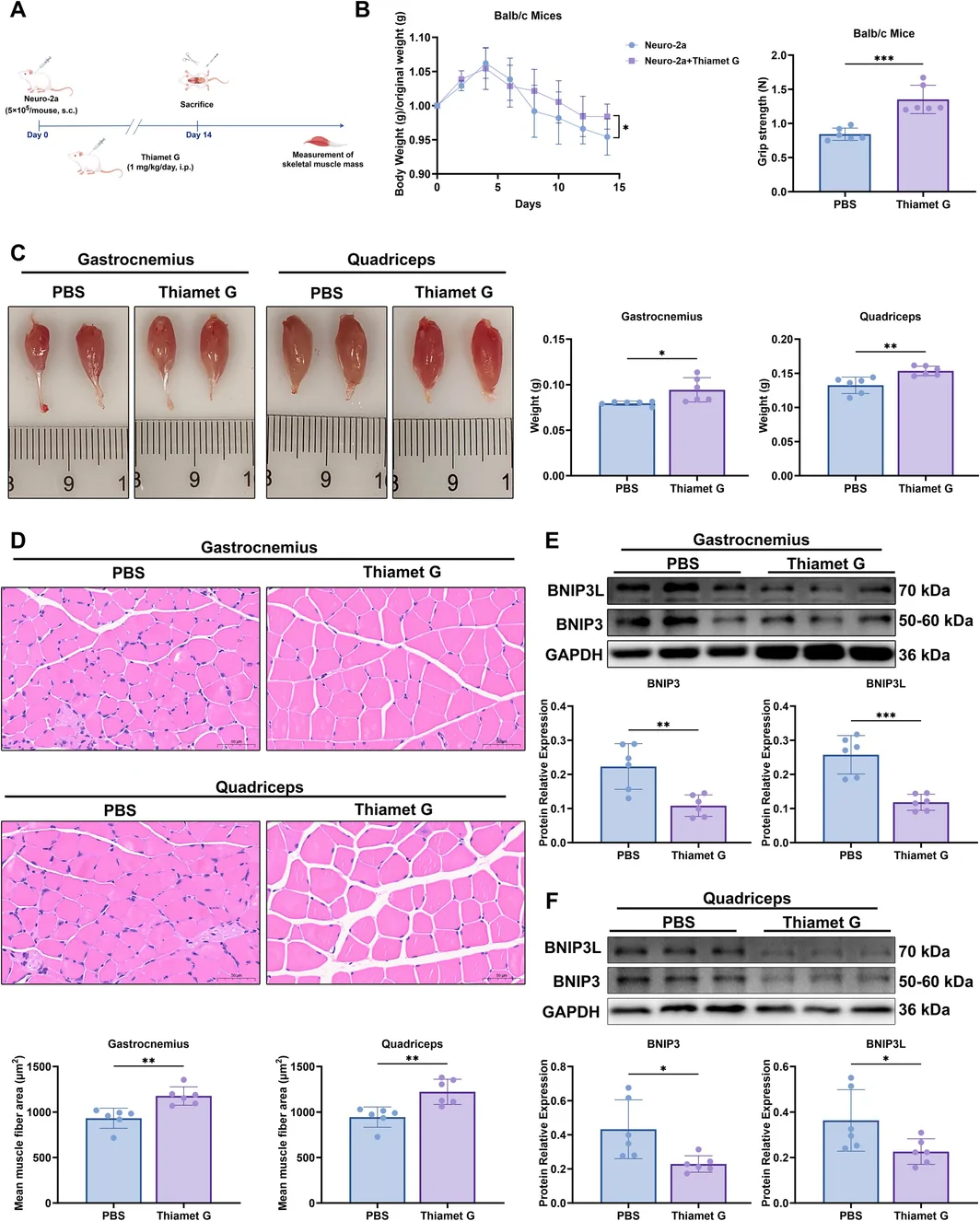
Thiamet G‐mediated alleviation of skeletal muscle atrophy in the NB mouse model via suppression of mitophagy. (A) Flowchart of the treatment process of Thiamet G on the NB mouse model. (B) Body weight changes and grip strength in the NB mouse model treated with PBS or Thiamet G (1 mg/kg) (*n* = 6 per group). (C) Gastrocnemius and quadriceps muscle images and weight quantification from NB mouse model treated with PBS or Thiamet G (1 mg/kg) (*n* = 6 per group). (D) Representative HE‐stained cross‐sectional images of gastrocnemius muscles and quantification of mean muscle fibre area of muscle fibres from NB mouse model treated with PBS or Thiamet G (1 mg/kg) (*n* = 6 per group). Scale bar: 50 μm. (E, F) Protein expression levels and grayscale quantification of BNIP3 and BNIP3L in gastrocnemius and quadriceps muscles of NB mouse model treated with PBS or Thiamet G (1 mg/kg) (*n* = 6 per group). Values are mean ± SD. Significance determined using unpaired *t*‐test. *p < 0.05, **p < 0.01, ***p < 0.001 and ****p < 0.0001.

## 4 Discussion

Skeletal muscle atrophy impairs prognosis and long‐term quality of life in NB survivors, yet its molecular mechanisms remain poorly understood. We explored the mechanism of NB‐associated muscle atrophy, focusing on the inflammatory mediator S100A9. S100A9 regulates protein O‐GlcNAcylation to influence atrophy, and O‐GlcNAcylation mediates atrophy by modulating mitophagy. This study reveals that S100A9 drives NB‐associated skeletal muscle atrophy by suppressing O‐GlcNAcylation, which triggers excessive mitophagy.

Systemic inflammation is a key factor of skeletal muscle atrophy [5]. S100A9 is an inflammatory mediator that is highly expressed in tumours of NB patients with bone marrow metastasis [9]. In many cancers, S100A9 can drive skeletal muscle atrophy [12]. Therefore, we investigated its role in NB and found that elevated serum S100A9 was able to inhibit O‐GlcNAcylation in skeletal muscle and thus induce skeletal muscle atrophy in both NB patients and NB mouse models. However, in vitro experiments revealed a concentration‐dependent biphasic effect. S100A9 promoted O‐GlcNAcylation at 125 ng/mL but inhibited it and induced myotube atrophy at higher concentrations. This biphasic effect likely involves receptor switching. Basal RAGE expression is low in healthy myofibres [16]. Under low S100A9, the ligand may preferentially engage TLR4, which could enhance cellular glucose uptake and HBP flux, increase the OGT substrate UDP‐GlcNAc and thereby favour elevated O‐GlcNAcylation [17]. Elevated S100A9 expression can upregulate RAGE in skeletal muscle, thereby promoting muscle atrophy [18, 19]. Thus, S100A9 binding may shift from TLR4 to RAGE, and sustained RAGE activation drives O‐GlcNAcylation downregulation and muscle atrophy.

The concentrations of recombinant S100A9 required for in vitro O‐GlcNAcylation inhibition far exceed physiological systemic levels in NB patients. Specifically, 2000 ng/mL was needed in C2C12 myotubes compared to approximately 400 to 700 pg/mL in patient serum. This difference of three to four orders of magnitude does not undermine the in vitro findings but warrants careful interpretation. Several factors may explain this apparent discrepancy. First, serum concentrations likely underestimate local tissue levels, as S100A9 accumulates in interstitial spaces or inflammatory sites to sufficient concentrations [20]. Second, we used recombinant S100A9 homodimer in vitro, whereas the dominant circulating species is the S100A8‐S100A9 heterodimer, whose calcium‐dependent tetramerization modulates inflammatory signalling, whereas distinct homodimeric configurations drive distinct immune outputs [21]. Third, acute 24‐h exposure in cell culture may require higher concentrations to produce observable effects, in contrast to chronic, sustained exposure in disease states in vivo [22]. Finally, non‐specific adsorption of recombinant proteins to the plastic surface of culture utensils may further reduce the effective concentration available to cells [23]. These factors do not compromise the in vitro findings but warrant caution when extrapolating to clinical settings. Future studies measuring local S100A9 in muscle tissue and assessing native heterodimer activity in vivo will bridge this translational gap.

PI3K/Akt/mTOR pathway can regulate the stability of OGT proteins. In normal cells, inhibition of AKT or mTOR phosphorylation can reduce OGT protein content and global O‐GlcNAcylation level [24]. Notably, PI3K/Akt/mTOR pathway is frequently downregulated in cancer‐associated skeletal muscle atrophy [25]. The above evidence suggests that inhibition of the PI3K/Akt/mTOR signalling axis may be a key mechanism leading to the reduction of OGT proteins in skeletal muscle cells and driving muscle atrophy. On the other hand, RAGE receptor activation can negatively regulate the PI3K/Akt/mTOR pathway. Binding of RAGE to its ligands can inhibit the level of PI3K/Akt/mTOR activation [26]. RAGE is upregulated in cancer‐related atrophy skeletal muscle and is closely related to the muscular atrophy phenotype [19]. In this study, a significant upregulation of RAGE receptors was observed in both NB mouse models and atrophied rectus abdominis muscle samples from NB patients. In vitro experiments further confirmed that activation of RAGE significantly inhibited O‐GlcNAcylation in myotubes. Taken together, we suggest that S100A9 binding to RAGE may inhibit the PI3K/Akt/mTOR signalling pathway in NB‐associated skeletal muscle atrophy, leading to increased degradation and decreased expression of OGT protein, resulting in the downregulation of O‐GlcNAcylation modification and abnormal mitophagy and ultimately promoting the occurrence of muscle atrophy. Of course, this intermediate signalling step has not been directly confirmed by complementation experiments in this study, and future mechanistic confirmation by specific activation or inhibition of the PI3K/Akt/mTOR pathway is needed.

Protein O‐GlcNAcylation modification is closely associated with the occurrence and development of skeletal muscle atrophy. Studies by Wei Yan et al. have shown that breast cancer can promote skeletal muscle atrophy by secreting miR‐122, which suppresses OGT protein expression [27]. Our study further validated this regulatory relationship in an NB model, indicating that downregulation of O‐GlcNAcylation modification may be universal across different tumour contexts. Moreover, reduced O‐GlcNAcylation is also observed in skeletal muscle atrophy induced by denervation, fasting and ageing, further supporting its involvement in the atrophy process [15]. However, not all types of skeletal muscle atrophy are accompanied by the downregulation of O‐GlcNAcylation modifications. For instance, in dexamethasone‐induced atrophy and atrophy associated with chronic obstructive pulmonary disease, the O‐GlcNAcylation modification is upregulated instead, highlighting the complexity of its regulatory effect [28, 29]. Therefore, clarifying the status of O‐GlcNAcylation in specific diseases facilitates targeted regulation of their pathological processes. This study reveals that skeletal muscle atrophy in the context of NB is accompanied by the downregulation of O‐GlcNAcylation, and this downregulation can directly induce atrophy, providing new insights into the mechanism of NB‐related muscle atrophy and the formulation of targeted therapeutic strategies.

An important finding of our study is the differential regulation of the core mitophagy machinery. Although OSMI‐1 treatment significantly upregulated Pink1, Bnip3 and Bnip3l mRNA levels, Prkn (Parkin) expression remained unchanged. This expression pattern suggests that O‐GlcNAcylation downregulation preferentially activates the BNIP3/BNIP3L receptor‐mediated mitophagy pathway, but not the classic PINK1/Parkin ubiquitin‐dependent cascade. In the classical pathway, PINK1 stabilization at the mitochondrial outer membrane recruits and activates Parkin for ubiquitination of outer membrane proteins, which in turn activates autophagy receptors [30]. In contrast, BNIP3 and BNIP3L act as direct receptors that bind LC3 at autophagosomes and are independent of the Parkin‐mediated ubiquitination process [31]. The stability of Parkin expression in our model suggests that the muscle wasting phenotype is mainly driven by mechanisms mediated by this receptor. Thus, our data refine the existing understanding of cancer‐associated muscle atrophy by highlighting O‐GlcNAcylation as the key switch that biases mitophagy toward the BNIP3/BNIP3L axis.

How O‐GlcNAcylation regulates receptor‐mediated mitophagy remains poorly understood across many cancers with muscle atrophy. Our study demonstrates that O‐GlcNAcylation downregulation promotes receptor‐mediated mitophagy in NB‐associated skeletal muscle atrophy. However, the precise mechanism by which O‐GlcNAcylation orchestrates this process remains unknown. A reasonable speculation is that this process involves reprogramming of transcription factor activity of known O‐GlcNAcylation modified substrates. FoxO3a is the core driver of skeletal muscle atrophy and autophagy [32]. It is a transcription factor for mitophagy genes such as Bnip3, and its transcriptional activity is dynamically regulated by O‐GlcNAcylation [33]. Knockdown of OGT can promote FOXO3A transcription and activity [33]. HIF‐1α is also a key factor driving BNIP3 and BNIP3L transcription [34]. However, unlike FoxO3a, it has been shown that the reduction of global O‐GlcNAcylation modification inhibits the O‐GlcNAcylation of HIF‐1α, which in turn promotes its degradation through the ubiquitin‐proteasome pathway, leading to a decrease in intracellular HIF‐1α protein content [35]. The downregulation of O‐GlcNAcylation in skeletal muscle was confirmed in both mouse models and human samples in this study. Based on the above results, we believe that in NB‐related atrophied skeletal muscle, the downregulation of O‐GlcNAcylation may not function by stabilizing HIF‐1α. On the contrary, the reduction of FoxO3a modification is more likely to release the restriction of FOXO3a modification, promote its transcription and transcriptional activity and then upregulate the expression of BNIP3 and BNIP3L, ultimately driving the enhancement of mitophagy.

Skeletal muscle is not a metabolically homogeneous tissue. There are significant differences in the composition of muscle fibre types and the bias of oxidative and glycolytic metabolism between trunk and limb muscles, which determine their sensitivity to catabolic stimuli [36]. In wasting diseases including cancer cachexia, fast‐contracting glycolytic fibres (Type II) are generally more susceptible, whereas slow‐contracting oxidized fibres (Type I) are somewhat shielded by PGC‐1α‐dependent programmes [37, 38]. In terms of fibre types, about 50% of the rectus abdominis muscle fibres were Type I and 50% were Type II [39]. The quadriceps was also about 50% Type I and 50% Type II [39]. Fifty‐nine percent of the gastrocnemius were Type I and 41% were Type II [40]. About 45% of psoas major muscles are Type I and 55% are Type II [39]. Based on the characteristics of Type II fibres being more susceptible to catabolic stimulation, psoas major muscle has the highest proportion of Type II fibres, which may atrophy earlier under the influence of tumours. In this study, patients with amyotrophy were mainly differentiated by the tPMA Z‐score, which was able to identify patients with muscle atrophy earlier given the higher proportion of Type II fibres in the psoas muscle and its susceptibility to tumour effects. The rectus abdominis muscle was chosen as the validation tissue in this study because it is easy to obtain in most abdominal cancer surgeries and has been one of the most used sampling sites for previous studies of cancer‐related muscle changes. However, whether the rectus abdominis muscle can fully represent the systemic muscle changes associated with cachexia has not been fully demonstrated. Nevertheless, most putative effects of malignant disease on skeletal muscle, including circulating tumour and inflammatory mediators, changes in hormone levels and deficiencies in energy and essential nutrients, are systemic. In addition, to ensure that the collected rectus abdominis muscle was indeed affected by the tumour and in atrophied state, rectus abdominis muscle samples from patients with significantly reduced tPMA Z‐score were used in all validation experiments in this study. Validation experiments have confirmed that these rectus abdominis muscles from atrophied patients do have atrophied phenotypes and corresponding molecular changes. In conclusion, we believe that rectus abdominis muscle can be used as a representative muscle tissue to verify skeletal muscle atrophy in NB patients.

Despite the mechanistic insights gained into NB‐induced skeletal muscle atrophy, this study has several limitations. First, the role of O‐GlcNAcylation in regulating mitophagy was not fully elucidated. The detailed mechanism by which O‐GlcNAcylation modulates mitophagy will be a subject of our future investigations. Second, regarding validation in human tissues, only rectus abdominis muscle samples were available, and the techniques for further confirming mitophagy were limited. This is because the clinical samples were obtained exclusively during NB surgery, making it unfeasible to collect gastrocnemius samples from the patients. This limited sample size consequently constrained the scope of additional validation experiments. Therefore, we plan to continue collecting rectus abdominis samples from patients to further expand the skeletal muscle sample bank for NB research. Third, although the present study demonstrated that S100A9 induced skeletal muscle atrophy in the NB mouse model by regulating OGT and O‐GlcNAcylation in male mice, future studies should explore whether these findings are sex‐specific by including female cohorts.

## 5 Conclusion

Our research reveals a novel pathway wherein elevated serum S100A9 in NB patients triggers skeletal muscle atrophy. The mechanism involves S100A9‐mediated suppression of protein O‐GlcNAcylation, which subsequently accelerates mitophagy, leading to muscle loss. This work underscores the critical role of aberrant O‐GlcNAcylation in driving mitophagy during atrophy and offers crucial insights for devising targeted therapeutic strategies to mitigate muscle wasting in NB survivors.

## Funding

This research was supported by the Health Research Project of Shanghai Pudong New Area Health Commission (PW2023D‐04) and Shanghai Hospital Development Center Foundation (SHDC22022306). Parts of Figure 8 was created by Figdraw (https://www.figdraw.com).

## Ethics Statement

This study was conducted in strict accordance with the ethical principles outlined in the Declaration of Helsinki. The collection and analysis of all human clinical data and samples involved were reviewed and approved by the Ethics Committee/Institutional Review Board of the Shanghai Children's Medical Center (SCMC), Shanghai Jiao Tong University School of Medicine (Approval Nos.: SCMCIRB‐K2023128‐1). All animal experimental procedures were conducted in strict compliance with the Guide for the Care and Use of Laboratory Animals issued by the National Institutes of Health (NIH). The research plan was approved by the Welfare and Ethics Committee of Laboratory Animals of Shanghai SINOGENE Life Science and Technology (Ethics Approval No.: XNG201‐2402‐001). The maximal tumour size permitted by the Welfare and Ethics Committee of Laboratory Animals of Shanghai SINOGENE Life Science and Technology committee was 15 mm. The maximal tumour size in this research was not exceeded.

## Consent

Informed consent was obtained from all participants' parents or legal guardians.

## Conflicts of Interest

The authors declare no conflicts of interest.

## Supporting information


**Figure S1:** Daily food intake of NB mouse models group and control group. (A) Daily food intake of NB mice model group and control group. (B) Total food intake of NB mice model group and control group. Values are mean ± SD. Significance determined using unpaired *t*‐test. *p < 0.05, **p < 0.01, ***p < 0.001 and ****p < 0.0001.
**Figure S2:** Muscle atrophy of the quadriceps in the NB mouse model. (A) Quadriceps muscle images and weight quantification from NB mouse model (*n* = 6) and its control mice (*n* = 6). (B) Representative haematoxylin and eosin (HE)‐stained cross‐sectional images of quadriceps muscle and quantification of mean muscle fibre area of muscle fibres from NB mouse model (*n* = 6) and control mice (*n* = 6). Scale bar: 50 μm. (C) Protein expression levels and grayscale quantification of TRIM63 and FBXO32 in quadriceps muscles of NB mouse model (*n* = 6) and control group (*n* = 6). Values are mean ± SD. Significance determined using unpaired *t*‐test. *p < 0.05, **p < 0.01, ***p < 0.001 and ****p < 0.0001.
**Figure S3:** Pro‐atrophy effect of S100A9 and anti‐atrophy effect of ABR‐238901. (A) Effects of S100A9 recombinant protein on body weight and grip strength in mice. (B) Effect of ABR‐238901 on body weight and grip strength of NB mouse model. Scale bar: 50 μm. Values are mean ± SD. Significance determined using unpaired *t*‐test. *p < 0.05, **p < 0.01, ***p < 0.001 and ****p < 0.0001.
**Figure S4:** S100A9‐mediated skeletal muscle atrophy in NB mice. (A) Quadriceps muscle images and weight quantification from mice treated with recombinant mouse S100A9 protein (20 μg/mouse; *n* = 6) or PBS control (*n* = 6), as well as NB mice treated with the S100A9 inhibitor ABR‐238901 (30 mg/kg; *n* = 6) or PBS control (*n* = 6). (B) Protein expression levels and grayscale quantification of TRIM63 and FBXO32 in gastrocnemius muscles from the following groups: mice treated with recombinant mouse S100A9 protein (20 μg/mouse; *n* = 6) or PBS control (*n* = 6), as well as NB mice treated with the S100A9 inhibitor ABR‐238901 (30 mg/kg; *n* = 6) or PBS control (*n* = 6). (C) Protein expression levels and grayscale quantification of TRIM63 and FBXO32 in quadriceps muscles from the following groups: mice treated with recombinant mouse S100A9 protein (20 μg/mouse; *n* = 6) or PBS control (*n* = 6), as well as NB mice treated with the S100A9 inhibitor ABR‐238901 (30 mg/kg; *n* = 6) or PBS control (*n* = 6). (D) Representative HE‐stained cross‐sectional images of gastrocnemius muscles and quantification of mean muscle fibre area of muscle fibres from following groups: mice treated with recombinant mouse S100A9 protein (20 μg/mouse; *n* = 6) or PBS control (*n* = 6), as well as NB mice treated with the S100A9 inhibitor ABR‐238901 (30 mg/kg; *n* = 6) or PBS control (*n* = 6). HE, haematoxylin and eosin. Scale bar: 50 μm. Values are mean ± SD. Significance determined using unpaired *t*‐test. *p < 0.05, **p < 0.01, ***p < 0.001 and ****p < 0.0001.
**Figure S5:** Downregulation of O‐GlcNAcylation modification in atrophic quadriceps muscles from the NB mouse model and recombinant mouse S100A9 protein‐treated mice. (A, B) Protein expression levels and grayscale quantification of OGT, OGA and overall O‐GlcNAcylation modification level in quadriceps muscles from the NB mouse models (*n* = 6) and control group (*n* = 6), as well as mouse treated with recombinant mouse S100A9 protein (20 μg/mouse; *n* = 6) or PBS control (*n* = 6). Values are mean ± SD. Significance determined using unpaired *t‐*test. *p < 0.05, **p < 0.01, ***p < 0.001 and ****p < 0.0001.
**Figure S6:** The OGT inhibitor OSMI‐1 and the OGA inhibitor Thiamet G significantly alter the global O‐GlcNAcylation levels in C2C12 myotubes. (A) CCK8 assay to investigate the effect of different concentrations of OSMI‐1 on the viability of C2C12 myotubes. (B) Protein expression levels of overall O‐GlcNAcylation modification in C2C12 myotubes treated with different concentrations of OSMI‐1. (C) CCK8 assay to investigate the effect of different concentrations of Thiamet G on the viability of C2C12 myotubes. (D) Protein expression levels of overall O‐GlcNAcylation modification level in C2C12 myotubes treated with different concentrations of Thiamet G. Values are mean ± SD. Significance determined using one‐way ANOVA followed by Dunnett's multiple comparisons test for comparisons among three or more groups. *p < 0.05, **p < 0.01, ***p < 0.001 and ****p < 0.0001.
**Figure S7:** Mitochondrial membrane potential detected by JC‐1 in OSMI‐1‐treated C2C12 myotubes. (A, B) JC‐1 staining for mitochondrial membrane potential in C2C12 myotubes treated with OSMI‐1. JC‐1 forms red fluorescent J‐aggregates (high membrane potential) or green fluorescent monomers (low membrane potential) (*n* = 3 per group). Values are mean ± SD. Significance determined using unpaired *t*‐test. *p < 0.05, **p < 0.01, ***p < 0.001 and ****p < 0.0001.
**Figure S8:** Effect of S100A9 on autophagic flux in C2C12 myotubes, assessed by Baf treatment. (A, B) Protein expression levels and grayscale quantification of BNIP3L, BNIP3, p62, the ratio of LC3‐II to LC‐I in C2C12 myotubes following DMSO (control; *n* = 3), recombinant protein of mouse S100A9 (20 μM; *n* = 3) and the combination of Bafilomycin A1 (Baf, 100 nM) with recombinant protein of mouse S100A9 (*n* = 3). Values are mean ± SD. Statistical significance was determined by one‐way ANOVA followed by Dunnett's multiple comparisons test for comparisons among three or more groups. *p < 0.05, **p < 0.01, ***p < 0.001 and ****p < 0.0001.
**Figure S9:** BNIP3 knockdown mitigates S100A9‐induced C2C12 myotube atrophy. (A) RT‐qPCR analysis of Bnip3 mRNA expression in C2C12 myotubes after BNIP3 knockdown (*n* = 3 per group). (B) Western Blot analysis of BNIP3 protein expression in C2C12 myotubes after BNIP3 knockdown (*n* = 3 per group). (C) Effect of BNIP3 knockdown on S100A9‐induced expression of atrophy‐related proteins (*n* = 3 per group). (D) Effect of BNIP3 knockdown on S100A9‐induced decrease in myotube diameter (*n* = 3 per group). Values are mean ± SD. Statistical significance was determined by one‐way ANOVA followed by Dunnett's multiple comparisons test for comparisons among three or more groups. *p < 0.05, **p < 0.01, ***p < 0.001 and ****p < 0.0001.
**Figure S10:** The atrophic quadriceps femoris muscle of mice and the atrophic rectus abdominis muscle of NB patients highly expressed mitophagy‐related proteins. (A, B) Protein expression levels and grayscale quantification of BNIP3L, BNIP3, p62, the ratio of LC3‐II to LC‐I in quadriceps muscles from the NB mouse models (*n* = 6) and control group (*n* = 6). (C, D) Protein expression levels and grayscale quantification of BNIP3L, BNIP3, p62, the ratio of LC3‐II to LC‐I in quadriceps muscles from mouse treated with recombinant mouse S100A9 protein (*n* = 6) or PBS (*n* = 6). (E, F) Protein expression levels and grayscale quantification of BNIP3L, BNIP3, p62, the ratio of LC3‐II to LC‐I in rectus abdominis muscles from NB patients with (*n* = 6) or without (*n* = 6) muscle atrophy. Values are mean ± SD. Significance determined using unpaired *t*‐test. *p < 0.05, **p < 0.01, ***p < 0.001 and ****p < 0.0001.
**Figure S11:** RAGE receptor expression in atrophied skeletal muscle. (A) RAGE receptor expression in atrophied rectus abdominis muscle (*n* = 6 per group). (B) RAGE receptor expression in atrophied gastrocnemius muscle (*n* = 6 per group). Values are mean ± SD. Significance determined using unpaired *t*‐test. *p < 0.05, **p < 0.01, ***p < 0.001 and ****p < 0.0001.
**Figure S12:** FPS‐ZM1‐mediated alleviation of skeletal muscle atrophy in the NB mouse model. (A) Body weight of mice in the four groups (Control, FPS‐ZM1, Neuro‐2a and Neuro‐2a + FPS‐ZM1) (*n* = 6 per group). (B) Grip strength of mice in the four groups (Control, FPS‐ZM1, Neuro‐2a and Neuro‐2a + FPS‐ZM1) (*n* = 6 per group). (C, D) Representative images and weights of the gastrocnemius and quadriceps muscles from the four groups (Control, FPS‐ZM1, Neuro‐2a and Neuro‐2a + FPS‐ZM1) (*n* = 6 per group). (E, F) Representative H&E staining images and myofiber cross‐sectional area of the gastrocnemius and quadriceps from the four groups (Control, FPS‐ZM1, Neuro‐2a and Neuro‐2a + FPS‐ZM1) (*n* = 6 per group). Values are mean ± SD. Statistical significance was determined by one‐way ANOVA followed by Dunnett's multiple comparisons test for comparisons among three or more groups. *p < 0.05, **p < 0.01, ***p < 0.001 and ****p < 0.0001.
**Figure S13:** FPS‐ZM1‐mediated restoration of skeletal muscle O‐GlcNAcylation and reduction of atrophy‐related protein expression in the NB mouse model. (A, B) Levels of overall O‐GlcNAcylation and atrophy‐related proteins in the gastrocnemius across the four groups (Control, FPS‐ZM1, Neuro‐2a and Neuro‐2a + FPS‐ZM1) (*n* = 6 per group). Values are mean ± SD. Statistical significance was determined by one‐way ANOVA followed by Dunnett's multiple comparisons test for comparisons among three or more groups. *p < 0.05, **p < 0.01, ***p < 0.001 and ****p < 0.0001.
**Figure S14:** Effect of Thiamet G on tumours in the NB mouse model. (A) Images of tumours from NB mice treated with PBS or Thiamet G (*n* = 6 per group). (B) Tumour weight in NB mice treated with PBS or Thiamet G (*n* = 6 per group). (C) Tumour volume in NB mice treated with PBS or Thiamet G (*n* = 6 per group). Values are mean ± SD. Significance determined using unpaired *t*‐test. *p < 0.05, **p < 0.01, ***p < 0.001 and ****p < 0.0001.

## Data Availability

The datasets used and analysed during the current study are available from the corresponding author on reasonable request.
