# Supplementary material for: S100A9 Mediates Neuroblastoma‐Associated Skeletal Muscle Atrophy via O‐GlcNAcylation Repression and Activate Mitophagy Activation
Source: J Cachexia Sarcopenia Muscle. 2026 Nov 5;17(6):e70419. doi: 10.1002/jcsm.70419 (PMC13735905; doi:10.1002/jcsm.70419)
Supplement: Supplementary file 1 [file JCSM-17-e70419-s001.docx]

**Supplementary Information for
S100A9 drives neuroblastoma-associated muscle wasting by suppressing O-GlcNAcylation to activate mitophagy**

Jianwei Lin**^1, #^**, Yuxin Zhang**^2, #^**, Qi Wang^3^, Jingchun Lv^1^, Bingqiang Han^4^, Dapeng Jiang**^1, *^**

**1.** **Materials and methods**

**1.1. Reagent for in vivo or in vitro treatment**

Non-tumor-bearing Balb/c mice were randomly divided into two groups (n=6 per group): a control group (treated with PBS) and an S100A9 (HY-P74583, MCE, State of New Jersey, USA) treatment group (20 µg per mouse, administered via intraperitoneal injection)[1]. The administration regimen consisted of intraperitoneal injections three times per week for three weeks. Tumor-bearing mice received intraperitoneal injections of the S100A9-specific inhibitor ABR-238901 (30 mg/kg, HY-141537, MCE, State of New Jersey, USA) , Thiamet G (1 mg/kg, HY-12588, MCE, State of New Jersey, USA) and FPS-ZM1 (1 mg/kg, HY-19370, MCE, State of New Jersey, USA) respectively, starting on the day of tumor inoculation and administered daily[1-3]. The control group received intraperitoneal injections of PBS. The body weight of the mice was measured every two days. Grip strength assay was captured by Rat and mouse grip tester. After treatment, the mice were euthanized in accordance with animal welfare guidelines. Immediately following euthanasia, bilateral gastrocnemius and quadriceps were excised, weighed, and photographed for morphological analysis.

After the differentiation of the C2C12 myotubules was completed, OSMI-1 (20 µM, HY-119738, MCE, State of New Jersey, USA), Thiamet G (1 µM, HY-12588, MCE, State of New Jersey, USA) were added respectively according to the experimental requirements for 24 hours, recombinant mouse S100A9 protein (2000 ng/ml) for 24 hours, recombinant mouse S100B protein (20 µg/ml, HY-P71276, MCE, State of New Jersey, USA) for 72 hours[4]. And treated with FPS-ZM1 (20 nM) for 24 hours [1]. Bafilomycin A1 (100 nM, HY-100558, MCE, State of New Jersey, USA) was administered 3 hours prior to the completion of OSMI-1 treatment and S100A9 treatment[5]. Observe the morphological changes of the C2C12 myotubes after treatment under a microscope.

**1.2. Transcriptomics** **analysis**

High-throughput transcriptomic analysis was performed at Shanghai OE Biotech Co., Ltd on RNA extracted from the gastrocnemius muscles of mice and from OSMI-1-treated C2C12 myotubes. The study included atrophic gastrocnemius muscle samples from Neuro-2a tumor-bearing mice (n =5), three normal gastrocnemius muscle samples from control mice (n =5), three OSMI-1-treated C2C12 myotube samples (n =3), and three DMSO-treated C2C12 myotube samples (as controls, n =3). Differential gene expression analysis was conducted using DESeq2, with the thresholds for identifying differentially expressed genes set at |log₂Fold Change| ≥ 1 and q-value < 0.05. All data analyses were conducted using OECloud tools (<https://cloud.oebiotech.com>), including differential expression Analysis and Gene Set Enrichment Analysis (GSEA) analysis.

**1.3. Small interfering RNA (siRNA) transfection**

To verify the function of the target gene in vitro, chemically synthesized small interfering RNA (siRNA) to knock down the target gene was used in C2C12 myotubes. Double-stranded siRNAs targeting the coding region of mouse Bnip3 with three independent sequences were designed as follows: siBNIP3-1: Sense 5'-GAGAAGAAGAGAAGUUGAATT-3', antisense 5'-UUCAACUUCUCUUCUUCTT-3'. siBNIP3-2: sense 5'-GGAUUGGUCAAGUCGACCATT-3', antisense 5'-UGGUCGACUUGACCAAUCCTT-3'. siBNIP3-3: sense 5'-GGACGAAGUAGCUCCAAGATT-3', antisense 5'-UCUUGGAGCUACUUCGUCCTT-3'. All siRNAs were prepared by Shanghai DNA Bioscience Co. Ltd. Synthesized in the annealed duplex form. Six days before transfection, C2C12 myoblasts were seeded at the appropriate density and replaced with differentiation medium to induce myotube formation. After 6 days of differentiation, cells confluent to form multinucleated myotubes, at which point siRNA transfection was performed according to Lipofectamine™ 3000 Reagent (Invitrogen/Thermo Fisher Scientific) instructions: The siRNA and Lipofectamine 3000 reagent were diluted in Opti-MEM™ serum-reduced medium, mixed gently, incubated at room temperature to form liposome-siRNA complexes, and then added into the cell culture medium drop by drop. The cells were collected at the corresponding time points after transfection, and the knockdown efficiency was verified at the mRNA and protein levels by RT-qPCR and Western blot. siBNIP3-2 knockdown was selected as the most efficient one and was used in all subsequent functional experiments.

**1.4. Western Blotting**

The tissue was ground in a tissue grinder and immersed in a RIPA Lysis Buffer (Beyotime, Shanghai, China) containing protease inhibitors (Beyotime, Shanghai, China). After grinding, it is lysed on ice for 30 minutes. For cells, they were washed three times with pre-cooled PBS and then lysed on ice for 30 minutes using a RIPA Lysis Buffer containing protease inhibitors. After centrifugation at 4°C and 12,000 × g for 5 minutes, the supernatant was boiled in SDS loading buffer (Beyotime, Shanghai, China). Equal amounts of proteins were separated by SDS-PAGE (Epizyme, Shanghai, China) and then transferred to a polyvinylidene fluoride membrane (Merck, Darmstadt, German). After sealing with rapid membrane sealing solution for 30 minutes, the primary antibody is added and incubated overnight at 4°C. Following three times TBST (Sangon, Shanghai, China) washes, the secondary antibody (Proteintech, Wuhan, China) and the blot were incubated at room temperature for two hours. Immunoblotting was performed using a Tanon Protein Imaging System 4600.

**1.5. JC-1 assay and immunofluorescence**

C2C12 cells were seeded into confocal dishes and induced to differentiate into myotubes. After the designated treatments, the mitochondrial membrane potential (ΔΨm) of C2C12 myotubes was assessed using a mitochondrial membrane potential assay kit with JC-1 (Beyotime, Shanghai, China). Specifically, 1 mL of JC-1 staining working solution was added to each dish and thoroughly mixed. The cells were then incubated in a 37°C cell culture incubator for 20 minutes protected from light. Following incubation, the cells were washed twice with pre-chilled JC-1 staining buffer (1×) to remove unbound dye. Subsequently, 2 mL of cell culture medium was added to each confocal dish, and the fluorescence signals indicative of mitochondrial membrane potential changes (red fluorescence for high ΔΨm, green fluorescence for low ΔΨm) were immediately observed and recorded using a laser scanning confocal microscope.

C2C12 cells were seeded onto glass coverslips placed in 24-well plates and induced to differentiate into myotubes. After differentiation and drug treatment, the cells were fixed with 4% paraformaldehyde solution (Beyotime, Shanghai, China). After fixation, cells were permeabilized with an immunostaining permeabilization buffer containing Triton X-100 (Beyotime, Shanghai, China) to enhance antibody penetration. Subsequently, the coverslips were blocked with immunostaining blocking buffer (Beyotime, Shanghai, China) to prevent non-specific binding. After blocking, diluted primary antibody was added directly and incubated overnight at 4°C. The next day, fluorescently labeled secondary antibody (ab150077 and ab150116, Abcam, Cambridge, UK) was then added and incubated in the dark. After secondary antibody incubation, the coverslips were followed by incubation with DAPI (Beyotime, Shanghai, China) staining solution in the dark to label cell nuclear. Finally, the coverslips were mounted with anti-fade mounting medium (Beyotime, Shanghai, China), and immediately observed under a laser scanning confocal microscope to capture and analyze the fluorescence signals of target proteins.

**1.6. Tissue hematoxylin-eosin staining and immunofluorescence**

Fresh tissues should be immediately immersed in an adequate amount of tissue fixative (Servicebio, Wuhan, China) after being removed from the body and fixed at room temperature for more than 24 hours. After the fixation was completed, the tissue was placed in a dehydration box and undergoes gradient alcohol dehydration and transparency treatment in sequence, followed by paraffin embedding. Trim the embedded wax blocked and continuously sliced them with a paraffin slicer. Set the slice thickness to 4 μm. Paraffin sections were dewaxed to water, followed by hematoxylin and eosin (Servicebio, Wuhan, China) staining in sequence, or operate in the same way as cellular immunofluorescence, then dehydration, neutral gum sealing, microscopic examination, image collection and analysis.

**1.7. Cell viability assay**

Cell viability was detected by Cell Counting Kit-8 (Beyotime, Shanghai, China). After C2C12 cells were cultured in 96-well plates and induced to differentiate into myotubes, they were treated with drugs at varying concentrations: OSMI-1 and Thiamet G at concentration gradients of 0, 1, 5, 10, 20, and 40 µM. All treatments were incubated at 37°C for 24 or 48 hours. To exclude solvent effects, control groups with corresponding DMSO concentrations were established for OSMI-1 concentration. After treatment, 100 µl of a pre-prepared detection solution (complete medium: CCK-8 reagent = 9:1, v/v) was added to each well, followed by incubation at 37°C in the dark for 2 hours. Finally, the absorbance of each well was measured at a wavelength of 450 nm using a microplate reader to assess cell viability.

**1.8. Elisa**

According to the standard operating procedures provided by the manufacturer, the concentration of S100A9 protein in serum samples was quantitatively detected using the following two ELISA kits respectively: Using the kit produced by Weiao Biotechnology determined the concentration of mouse S100A9 protein (EM30461M) in mouse serum (EH10421M). The concentration of human S100A8 (EH10421M), S100A9 (EH10633M), and S100A8/S100A9 (EH11320M) in human serum was determined using the same brand reagent kit. All operations were carried out strictly in accordance with the steps specified in the kit manual, including sample addition, incubation, washing, color development and determination of absorbance value at 450 nm wavelength, and the concentration of the target protein was calculated through the standard curve.

**1.9. Transmission electron microscope (TEM)**

Fresh gastrocnemius muscle tissue from mice was extracted, with a tissue volume not exceeding 1mmx1mmx1mm. Quickly add the electron microscope fixative (Servicebio, Wuhan, China) and fix at 4℃ for 4 hours. Then the fixed samples were sent to BaiQianDu Biotechnology for TEM examination to observe the mitophagy of the gastrocnemius and quadriceps.

**1.10. Reverse Transcription Quantitative Real-time Polymerase Chain Reaction (RT-qPCR)**

After extracting total RNA from tissues or cells using the Trizol (Thermo Fisher, Massachusetts, USA) method, it was reverse transcribed into cDNA by the Evo M-MLV RT Mix Kit (Accurate, Guangzhou, China). Furthermore, amplification was carried out on a real-time fluorescence quantitative PCR instrument using the SYBR Green dye (Accurate, Guangzhou, China) method (the reaction system contained 10 μl of SYBR Green, 0.4 μl each of the upstream and downstream primers, 5 μl of cDNA, and 4.2 μl of ddH₂O), and the specificity of the product was verified by the melting curve. The relative expression levels of FBXO32, TRIM63, Bnip3, Bnipl Pink1 and Prkn genes were ultimately calculated using the 2 ^-ΔΔCt^ method with actin as the internal reference. The primers sequences were as follows: Human ACTIN mRNA forward primer: 5′GACCTGTACGCCAACACAGT3′, reverse primer: 3′CTCAGGAGGAGCAATGATCT5′. Human TRIM63 mRNA forward primer: 5′CTTGACTGCCAAGCAACTC3′. reverse primer: 3′TCCTCATCTGTCCCAAAGTC5′. Human FBXO32 mRNA forward primer: 5′CAGCAGTTACTGCAACAAGG3′, reverse primer: 3′CCCAGGGTGCAATATCCA5′. Mouse Actin mRNA forward primer: 5′GGCTGTATTCCCCTCCATCG3′, reverse primer: 5′CCAGTTGGTAACAATGCCATGT3′. Mouse Bnip3 mRNA forward primer: 5′CTGGACGAAGTAGCTCCAAGA3′. reverse primer: 3′ACAGAGTGCTGTTTTTCTCGC5′. Mouse Bnip3l mRNA forward primer: 5′GAGGCAGTTCGCACTGTGA3′. reverse primer: 3′ACCAGTCTGATACCCAGTCTG5′. Mouse Prkn mRNA forward primer: 5′GAGGTCGATTCTGACACCAGC3′. reverse primer: 3′CCGGCAAAAATCACACGCAG5′. Mouse Pink1 mRNA forward primer: 5′TTCTTCCGCCAGTCGGTAG3′. reverse primer: 3′CTGCTTCTCCTCGATCAGCC5′.

**1.11. Antibody**

TRIM63 (Abclonal #A3101, Wuhan, China), O-GlcNAc (CST #82332S, Massachusetts USA), OGT (Proteintech #11576-2-AP, Chicago, USA), OGA (Proteintech Group, 14711-1-AP), BNIP3L (Proteintech #12986-1-AP, Chicago, USA), BNIP3 (Proteintech #68091-1-Ig, Chicago, USA), TOMM20 (Proteintech #11802-1-AP, Chicago, USA), TLR4 (Proteintech #19811-1-AP, Chicago, USA), RAGE (Proteintech#66833-1-Ig, Chicago, USA), FBXO32 (Proteintech #67172-1-Ig, Chicago, USA), SQSTM1/p62 (Zenbio, #380612, Chengdu, China). LC3A/B (Zenbio #306019, Chengdu, China).

**1.12. Analysis of public databases**

The GeneCards human gene database (<https://www.genecards.org/>) was used to collect genes related to cachexia[6]. The Human Protein Atlas database (<https://www.proteinatlas.org/>) was used used to screen out secreted proteins in the blood[7]. The R2: Genomics Analysis and Visualization Platform (<http://r2.amc.nl>) was used to analyze the impact of S100A9 expression level on the prognosis of NB patients. The bioinformatics Online analysis website of ASSISTANT for Clinical Bioinformatics database (<https://www.aclbi.com/static/index.html#/>) was used to analyze the differential genes of NB in public databases (GSE49710, GSE112447)[8, 9].

**References**

1.	Zhang, Q., et al., *PGAM5 interacts with and maintains BNIP3 to license cancer-associated muscle wasting.* Autophagy, 2024. **20**(10): p. 2205-2220.

2.	Suenaga, T., et al., *O-GlcNAcase Inhibitor Improves Denervation-Induced Muscle Atrophy in Mice.* J Cachexia Sarcopenia Muscle, 2025. **16**(5): p. e70066.

3.	Santoro, T., et al., *Glucocorticoids decrease the numbers and activation of mast cells by inducing the transactivation receptors of AGEs.* J Leukoc Biol, 2019. **105**(1): p. 131-142.

4.	Chiappalupi, S., et al., *Targeting RAGE prevents muscle wasting and prolongs survival in cancer cachexia.* J Cachexia Sarcopenia Muscle, 2020. **11**(4): p. 929-946.

5.	Summers, C.M. and R.J. Valentine, *Acute Heat Exposure Alters Autophagy Signaling in C2C12 Myotubes.* Front Physiol, 2019. **10**: p. 1521.

6.	Stelzer, G., et al., *The GeneCards Suite: From Gene Data Mining to Disease Genome Sequence Analyses.* Curr Protoc Bioinformatics, 2016. **54**: p. 1 30 1-1 30 33.

7.	Uhlen, M., et al., *The human secretome.* Sci Signal, 2019. **12**(609).

8.	Yu, G., et al., *clusterProfiler: an R package for comparing biological themes among gene clusters.* OMICS, 2012. **16**(5): p. 284-7.

9.	Huang, H., et al., *Exploring Ferroptosis-Associated Gene Signatures as Diagnostic and Therapeutic Targets for Sepsis-Induced Cardiomyopathy.* Cureus, 2024. **16**(5): p. e60439.

**Supplementary Figures**

**Figure S1. Daily food intake of NB mouse models group and control group.**

(A) Daily food intake of NB mice model group and control group. (B) Total food intake of NB mice model group and control group. Values are mean ± SD. Significance determined using unpaired t test. *p < 0.05, **p < 0.01, ***p < 0.001 and ****p < 0.0001.

**Figure S2. Muscle atrophy of the quadriceps in the NB mouse model.**

**(A)** Quadriceps muscle images and weight quantification from NB mouse model (n=6) and its control mice (n=6). **(B)** Representative Hematoxylin and Eosin (HE)-stained cross-sectional images of quadriceps muscle and quantification of mean muscle fiber area of muscle fibers from NB mouse model (n=6) and control mice (n=6). Scale bar: 50 μm. **(C)** Protein expression levels and grayscale quantification of TRIM63 and FBXO32 in quadriceps muscles of NB mouse model (n=6) and control group (n=6). Values are mean ± SD. Significance determined using unpaired *t* test. **p* < 0.05, ***p* < 0.01, ****p* < 0.001 and *****p* < 0.0001.

**Figure S3. Pro-atrophy effect of S100A9 and anti-atrophy effect of ABR-238901.**

**(A)** Effects of S100A9 recombinant protein on body weight and grip strength in mice. **(B)** Effect of ABR-238901 on body weight and grip strength of NB mouse model. Scale bar: 50 μm. Values are mean ± SD. Significance determined using unpaired t test. *p < 0.05, **p < 0.01, ***p < 0.001 and ****p < 0.0001.

**Figure S4. S100A9-mediated skeletal muscle atrophy in NB mice. (A)** Quadriceps muscle images and weight quantification from mice treated with recombinant mouse S100A9 protein (20 μg/mouse; n=6) or PBS control (n=6), as well as NB mice treated with the S100A9 inhibitor ABR-238901 (30 mg/kg; n=6) or PBS control (n=6). **(B)** Protein expression levels and grayscale quantification of TRIM63 and FBXO32 in gastrocnemius muscles from the following groups: mice treated with recombinant mouse S100A9 protein (20 μg/mouse; n=6) or PBS control (n=6), as well as NB mice treated with the S100A9 inhibitor ABR-238901 (30 mg/kg; n=6) or PBS control (n=6). **(C)** Protein expression levels and grayscale quantification of TRIM63 and FBXO32 in quadriceps muscles from the following groups: mice treated with recombinant mouse S100A9 protein (20 μg/mouse; n=6) or PBS control (n=6), as well as NB mice treated with the S100A9 inhibitor ABR-238901 (30 mg/kg; n=6) or PBS control (n=6). **(D)** Representative HE-stained cross-sectional images of gastrocnemius muscles and quantification of mean muscle fiber area of muscle fibers from following groups: mice treated with recombinant mouse S100A9 protein (20 μg/mouse; n=6) or PBS control (n=6), as well as NB mice treated with the S100A9 inhibitor ABR-238901 (30 mg/kg; n=6) or PBS control (n=6). HE, Hematoxylin and Eosin. Scale bar: 50 μm. Values are mean ± SD. Significance determined using unpaired *t* test. **p* < 0.05, ***p* < 0.01, ****p* < 0.001 and *****p* < 0.0001.

**Figure S5. Downregulation of O-GlcNAcylation modification in atrophic quadriceps muscles from the NB mouse model and recombinant mouse S100A9 protein-treated mice. (A-B)** Protein expression levels and grayscale quantification of OGT, OGA and overall O-GlcNAcylation modification level in quadriceps muscles from the NB mouse models (n=6) and control group (n=6), as well as mouse treated with recombinant mouse S100A9 protein (20 μg/mouse; n=6) or PBS control (n=6). Values are mean ± SD. Significance determined using unpaired *t* test. **p* < 0.05, ***p* < 0.01, ****p* < 0.001 and *****p* < 0.0001.

**Figure S6. The OGT inhibitor OSMI-1 and the OGA inhibitor Thiamet G significantly alter the global O-GlcNAcylation levels in C2C12 myotubes. (A)** CCK8 assay to investigate the effect of different concentrations of OSMI-1 on the viability of C2C12 myotubes. **(B)** Protein expression levels of overall O-GlcNAcylation modification in C2C12 myotubes treated with different concentrations of OSMI-1. **(C)** CCK8 assay to investigate the effect of different concentrations of Thiamet G on the viability of C2C12 myotubes. **(D)** Protein expression levels of overall O-GlcNAcylation modification level in C2C12 myotubes treated with different concentrations of Thiamet G. Values are mean ± SD. Significance determined using one-way ANOVA followed by Dunnett's multiple comparisons test for comparisons among three or more groups. **p* < 0.05, ***p* < 0.01, ****p* < 0.001 and *****p* < 0.0001.

**Figure S7. Mitochondrial membrane potential detected by JC-1 in OSMI-1-treated C2C12 myotubes.** **(A-B)** JC-1 staining for mitochondrial membrane potential in C2C12 myotubes treated with OSMI-1. JC-1 forms red fluorescent J-aggregates (high membrane potential) or green fluorescent monomers (low membrane potential) (n=3 per group). Values are mean ± SD. Significance determined using unpaired t test. *p < 0.05, **p < 0.01, ***p < 0.001 and ****p < 0.0001.

**Figure S8. Effect of S100A9 on autophagic flux in C2C12 myotubes, assessed by Baf treatment.** **(A-B)** Protein expression levels and grayscale quantification of BNIP3L, BNIP3, p62, the ratio of LC3-II to LC-I in C2C12 myotubes following DMSO (control; n=3), recombinant protein of mouse S100A9 (20 μM; n=3) and the combination of Bafilomycin A1 (Baf, 100 nM) with recombinant protein of mouse S100A9 (n=3). Values are mean ± SD. Statistical significance was determined by one-way ANOVA followed by Dunnett's multiple comparisons test for comparisons among three or more groups. *p < 0.05, **p < 0.01, ***p < 0.001 and ****p < 0.0001.

**Figure S9. BNIP3 knockdown mitigates S100A9-induced C2C12 myotube atrophy. (A)** RT-qPCR analysis of Bnip3 mRNA expression in C2C12 myotubes after BNIP3 knockdown (n=3 per group). **(B)** Western Blot analysis of BNIP3 protein expression in C2C12 myotubes after BNIP3 knockdown (n=3 per group). **(C)** Effect of BNIP3 knockdown on S100A9-induced expression of atrophy-related proteins (n=3 per group). **(D)** Effect of BNIP3 knockdown on S100A9-induced decrease in myotube diameter (n=3 per group). Values are mean ± SD. Statistical significance was determined by one-way ANOVA followed by Dunnett's multiple comparisons test for comparisons among three or more groups. *p < 0.05, **p < 0.01, ***p < 0.001 and ****p < 0.0001.

**Figure S10. The atrophic quadriceps femoris muscle of mice and the atrophic rectus abdominis muscle of NB patients highly expressed mitophagy-related proteins. (A-B)** Protein expression levels and grayscale quantification of BNIP3L, BNIP3, p62, the ratio of LC3-II to LC-I in quadriceps muscles from the NB mouse models (n=6) and control group (n=6). **(C-D)** Protein expression levels and grayscale quantification of BNIP3L, BNIP3, p62, the ratio of LC3-II to LC-I in quadriceps muscles from mouse treated with recombinant mouse S100A9 protein (n=6) or PBS (n=6). **(E-F)** Protein expression levels and grayscale quantification of BNIP3L, BNIP3, p62, the ratio of LC3-II to LC-I in rectus abdominis muscles from NB patients with (n=6) or without (n=6) muscle atrophy. Values are mean ± SD. Significance determined using unpaired *t* test. **p* < 0.05, ***p* < 0.01, ****p* < 0.001 and *****p* < 0.0001.

**Figure S11. RAGE receptor expression in atrophied skeletal muscle. (A)** RAGE receptor expression in atrophied rectus abdominis muscle (n=6 per group). **(B)** RAGE receptor expression in atrophied gastrocnemius muscle (n=6 per group). Values are mean ± SD. Significance determined using unpaired *t* test. **p* < 0.05, ***p* < 0.01, ****p* < 0.001 and *****p* < 0.0001.

**Figure S12. FPS-ZM1-mediated alleviation of skeletal muscle atrophy in the NB mouse model.** **(A)** Body weight of mice in the four groups (Control, FPS-ZM1, Neuro-2a, and Neuro-2a+FPS-ZM1) (n=6 per group). **(B)** Grip strength of mice in the four groups (Control, FPS-ZM1, Neuro-2a, and Neuro-2a+FPS-ZM1) (n=6 per group). **(C-D)** Representative images and weights of the gastrocnemius and quadriceps muscles from the four groups (Control, FPS-ZM1, Neuro-2a, and Neuro-2a+FPS-ZM1) (n=6 per group). **(E-F)** Representative H&E staining images and myofiber cross-sectional area of the gastrocnemius and quadriceps from the four groups (Control, FPS-ZM1, Neuro-2a, and Neuro-2a+FPS-ZM1) (n=6 per group). Values are mean ± SD. Statistical significance was determined by one-way ANOVA followed by Dunnett's multiple comparisons test for comparisons among three or more groups. *p < 0.05, **p < 0.01, ***p < 0.001 and ****p < 0.0001.

**Figure S13. FPS-ZM1-mediated restoration of skeletal muscle O-GlcNAcylation and reduction of atrophy-related protein expression in the NB mouse model. (A-B)** Levels of overall O-GlcNAcylation and atrophy-related proteins in the gastrocnemius across the four groups (Control, FPS-ZM1, Neuro-2a, and Neuro-2a+FPS-ZM1) (n=6 per group). Values are mean ± SD. Statistical significance was determined by one-way ANOVA followed by Dunnett's multiple comparisons test for comparisons among three or more groups. *p < 0.05, **p < 0.01, ***p < 0.001 and ****p < 0.0001.

**Figure S14. Effect of Thiamet G on tumors in the NB mouse model.** **(A)** Images of tumors from NB mice treated with PBS or Thiamet G (n=6 per group). **(B)** Tumor weight in NB mice treated with PBS or Thiamet G (n=6 per group). **(C)** Tumor volume in NB mice treated with PBS or Thiamet G (n=6 per group). Values are mean ± SD. Significance determined using unpaired *t* test. **p* < 0.05, ***p* < 0.01, ****p* < 0.001 and *****p* < 0.0001.
